# Bioactive Polycyclic Quinones from Marine *Streptomyces* sp. 182SMLY

**DOI:** 10.3390/md14010010

**Published:** 2016-01-06

**Authors:** Ying Liang, Xin Xie, Lu Chen, Shilun Yan, Xuewei Ye, Komal Anjum, Haocai Huang, Xiaoyuan Lian, Zhizhen Zhang

**Affiliations:** 1 Ocean College, Zhejiang University, Hangzhou 310058, China; lynne3665@163.com (Y.L.); chenlu0310@sina.com (L.C.); slyanphd@163.com (S.Y.); moshangmowei@163.com (X.Y.); komalazam@ymail.com (K.A.); hchuang@zju.edu.cn (H.H.); 2 College of Pharmaceutical Sciences, Zhejiang University, Hangzhou 310058, China; 21319046@zju.edu.cn

**Keywords:** marine bacterium *Streptomyces* sp. 182SMLY, *N*-acetyl-*N*-demethylmayamycin, streptoanthraquinone A, bioactivities against glioma cells and bacteria

## Abstract

Chemical investigation of the cultures of marine *Streptomyces* sp. 182SMLY led to the discovery of two new polycyclic anthraquinones, which were elucidated as *N*-acetyl-*N*-demethylmayamycin (**1**) and streptoanthraquinone A (**2**) based on the extensive spectroscopic analysis including 2D NMR, HRESIMS, and an electronic circular dichroism (ECD) calculation. Both anthraquinones remarkably suppressed the proliferation of four different glioma cell lines with IC_50_ values in a range from 0.5 to 7.3 μM and induced apoptosis in the glioma cells. The ratios of IC_50_ for normal human astrocytes to IC_50_ for glioma cells were 6.4–53 for **1** and >14–31 for **2**. *N*-acetyl-*N*-demethylmayamycin (**1**) also inhibited the growth of methicillin-resistant *Staphylococcus aureus* with MIC 20.0 μM.

## 1. Introduction

Gliomas represent 80% of primary malignant brain tumors and remain a serious health problem despite advances in a standard treatment regimen of surgical resection followed by radiation and chemotherapy [1,2]. While chemotherapy has played an important role in the treatment and prevention of cancer, very few drugs have been approved for treating gliomas including temozolomide (TMZ), carmustine, lomustine, and bevacizumab [3]. Furthermore, only TMZ has been independently used for the treatment of gliomas, and the efficacy of TMZ and other current anti-glioma drugs remains unsatisfactory [3]. Therefore, there is an urgent need to discover lead compounds for the development of novel anti-glioma drugs. Marine-derived natural products are important sources for the discovery of new anticancer drug leads [4,5,6].

During the course of our ongoing project for the discovery of novel antibacterial and anti-tumor natural products from marine organisms [7,8,9,10,11,12], the cultures of marine bacterium strain 182SMLY isolated from a sediment sample was found to inhibit the proliferation of glioma cells. The 16S rDNA gene sequence of strain 182SMLY completely (100% identity for a 1376 bp stretch of sequence) matched those of several *Streptomyces* strains (Table S1), including *S. griseus* CB00830, *S. pluricolorescens* 999, *S. tricolor* cfcc3055, *S. sporovirgulis* L0801, *S.* sp. A15Ydz-AH, *S.* sp. HBUM74775, and *S. sporovirgulis* TGNBSA5 in the GenBank database. Previous studies showed that actinomycete *S. griseus* produced diversified secondary metabolites mainly including macrolides [13,14], β-lactams [15,16], quinones [17], aminoglycosides [18], diterpenoids [19], and phenazines [20], while *S. pluricolorescens* produced *C*-glycosidic quinones [21].

**Figure 1:**
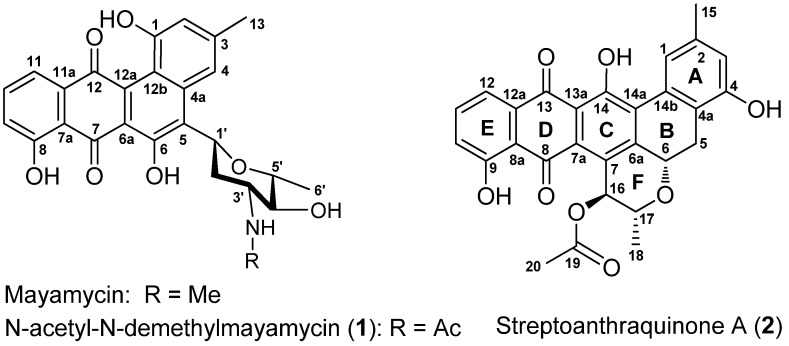
Structures of compounds **1** and **2**.

In this study, two novel polycyclic quinones of *N*-acetyl-*N*-demethylmayamycin (**1**) and streptoanthraquinone A (**2**) (Figure 1) were isolated from the cultures of strain 182SMLY by using column chromatographic fractionation, followed by HPLC purification. We report herein the isolation and culture of strain 182SMLY, the structural elucidation of the isolates, and their inhibitory activities against the proliferation of glioma cells and the growth of methicillin-resistant *Staphylococcus aureus* and *Escherichia coli*.

## 2. Results and Discussion

Strain 182SMLY was isolated from a sediment sample collected from the East China Sea and classified as a *Streptomyces* species based on the result of the 16S rDNA sequence analysis. Cultures of this bacterium were grown in the Gause’s liquid medium (30 L) and then extracted by organic solvents. The extract was fractionated by column chromatography, followed by HPLC purification, to yield two anthraquinones.

### 2.1. Structure Elucidation

**Figure 2:**
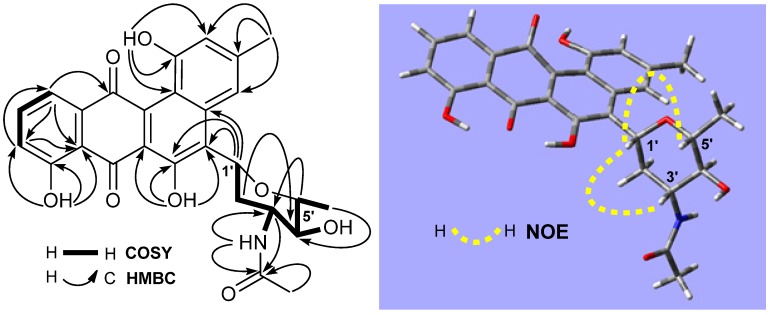
^1^H–^1^H COSY, key HMBC and NOE correlations of *N*-acetyl-*N*-demethylmayamycin (**1**).

**Figure 3:**
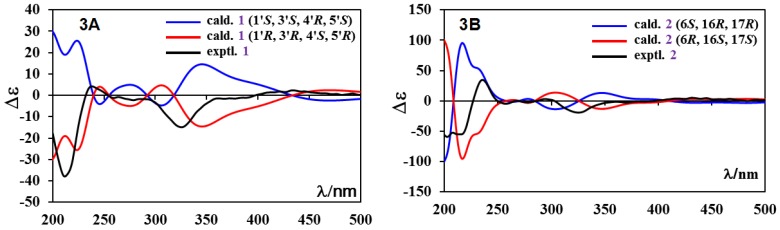
The experimental and calculated ECD spectra of (**A**) *N*-acetyl-*N*-demethylmayamycin (**1**) and (**B**) streptoanthraquinone A (**2**).

Compound **1** was obtained as a brown amorphous solid and had a molecular formula of C_27_H_25_NO_8_, as determined by its HRESIMS at *m/z* 514.1457 [M + Na]^+^ (calcd for C_27_H_25_NNaO_8_, 514.1478). Its UV absorptions at 328 and 443 nm are similar to those of mayamycin, belonging to the angucycline group [22]. The quinone group in **1** was suggested by two carbonyl signals at δ 191.6 and 184.6 (Table 1). HMBC correlations of three singlet signals at δ_H_ 12.26 (1H, OH-6), 11.36 (1H, OH-8), and 10.26 (1H, OH-1) with the three carbon signals at δ_C_ 152.1 (C-6), 160.2 (C-8), and 155.2 (C-1) indicated the presence of three hydroxy groups. The region (δ 6.5–8.0) of ^1^H-NMR spectrum showed five aromatic proton signals: two appeared at δ 6.64 (1H, brs) and 7.89 (1H, brs) and were assigned to H-2 and H-4; two were double doublets at δ 7.30 (1H, 7.5, 1.1 Hz) and 7.43 (1H, 7.5, 1.1 Hz) and were assigned to H-9 and H-11; and one was a triplet at δ 7.76 (1H, 7.5 Hz) and was assigned to H-10. In addition, the signals (δ_C_ 22.3; δ_H_ 2.40, 3H, s) for CH_3_-13 were also observed. From these NMR data, it was altogether concluded that both **1** and mayamycin had the same dehydrorabelomycin [23] as their quinone skeletons. Further NMR spectroscopic interpretation indicated that the structural difference between the two compounds was their amino sugar parts; *i.e.*, the *N*-CH_3_ group in mayamycin was replaced by the *N*-acetyl group in **1**. Therefore, the amino sugar in **1** was assigned as 2,3,6-trideoxy-3-acetylaminopyran, which was further confirmed by COSY correlations in combination with HMBC information, shown in Table 1 and Figure 2. The linkage of this amino sugar at C-5 was established by HMBC correlations of H-1′ (δ 5.44) with C-4a (δ 137.6), C-5 (δ 124.2), and C-6 (δ 152.1). The ^3^*J*_H4′-H5′_ coupling constant (9.1 Hz) and the strong NOE correlations of H-1′ (δ 5.44) with H-3′ (δ 3.85) and H-5′ (δ 3.40) (Figure 2) in the NOESY spectrum indicated the same orientation for H-1′, H-3′, and H-5′, suggesting that the relative configuration of this amino is 1′*R*, 3′*R*, 4′*S*, 5′*R* (22). In order to assign the absolute configuration, the theoretical calculation of ECD spectrum of **1** was carried out. Two preferred conformers (Figures S43 and S44) of (1′*R*,3′*R*,4′*S*,5′*R*)-**1** were yielded after an OPLS (optimized potentials for liquid simulations) conformational search followed by DFT (density functional theory) optimization at the B3LYP/6-31+G(d) level. The ECD spectra of both conformers were calculated using the TDDFT (time-dependent density functional theory) method at the B3LYP-SCRF/6-31+G(d) level in methanol. The Boltzmann-weighted ECD spectrum of the conformer (1′*R*,3′*R*,4′*S*,5′*R*)-**1** showed good agreement with the experimental curve of **1** (Figure 3A). Thus, the absolute configuration of **1** was assigned as (1′*R*,3′*R*,4′*S*,5′*R*). The full ^1^H and ^13^C assignments (Table 1) of **1** were made based on the COSY, HSQC, HMBC, and NOESY spectroscopic interpretations. Compound **1** was determined as *N*-acetyl-*N*-demethylmayamycin, a new analogue of mayamycin (22). Cytotoxic and antibacterial mayamycin was previously isolated from *Streptomyces* sp. strain HB202, a symbiotic bacterium with the marine sponge *Halichondria panicea*, and is the first example in the angucycline class with a *C*-glycoside at the C-5 of the quinone skeleton [22]. Although a robust regio- and stereocontrolled route for the synthesis of mayamycin scaffold has been recently developed [24], the total synthesis of mayamycin has not achieved yet [24,25] and is still underway [24]. To the best of our knowledge, *N*-acetyl-*N*-demethylmayamycin (**1**) is the first reported addition to this class of mayamycin.

**Table 1:** ^13^C- and ^1^H-NMR Data of *N*-acetyl-*N*-demethylmayamycin (**1**) (in DMSO-*d*_6_).

No.	δ_C_, Type	δ_H_ (*J* = Hz)	HMBC	No.	δ_C_, Type	δ_H_ (*J* = Hz)	HMBC
1	155.2, C			12a	137.7 ^a^, C		
2	111.5, CH	6.64, brs	1, 4, 12b, 13	12b	115.4, C		
3	141.0, C			13	22.3, CH_3_	2.40, s	2, 3, 4
4	115.4, CH	7.89, brs	2, 5, 12b, 13	1′	71.0, CH	5.44, dd (12.1, 2.3)	4a, 5, 6
4a	137.6 ^a^, C			2′	36.0, CH_2_	1.86, m; 2.10, 1H, m	1′, 3′, 4′
5	124.2, C			3′	52.5, CH	3.85, m	7′
6	152.1, C			4′	74.2, CH	3.20, m	3′
6a	117.6, C			5′	77.7, CH	3.40, dd (9.1, 6.1)	3′
7	191.6, C			6′	18.6, CH_3_	1.27, d (6.1)	4′, 5′
7a	115.6, C			7′	169.2, C		
8	160.2, C			8′	22.3, CH_3_	1.80, s	7′
9	122.9, CH	7.30, dd (7.5, 1.1)	7a, 8, 11	1-OH		10.26, s	1, 2, 12b
10	137.5, CH	7.76, t (7.5)	8, 11, 11a	6-OH		12.26, s	5, 6, 6a
11	117.9, CH	7.43, dd (7.5, 1.1)	7a, 9, 12	8-OH		11.36, s	7a, 8, 9
11a	136.6, C			3′-NH		7.87, d (8.2)	3′, 7′
12	184.6, C			4′-OH		5.02, d (5.1)	

**^a^** The data with the same label in each column may be interchanged.

Compound **2**, a dark brown amorphous powder, had a molecular formula of C_28_H_22_O_8_ deduced from its HRESIMS data at *m/z* [M + Na]^+^ 509.1233 (calcd for C_28_H_22_NaO_8_, 509.1212) and ^13^C-NMR data (Table 2). The prelimirary NMR spectroscopic interpretation indicated that compound **2** contained the moieties of a quinone group (δ_C_ 193.5, 189.6), a carbonyl (δ_C_ 170.3), three hydroxyls, 18 aromatic carbons, three oxymethines, three methyls, and a methylene. These data suggested that **2** may have a dihydrobenzo[*a*]naphthacenequinone skeleton, which usually has five rings A, B, C, D, and E (Figure 1) [26]. More detailed NMR data from ^1^H, ^13^C, ^1^H–^1^H COSY, HSQC, HMBC, and NOESY spectra and an ECD calculation enabled the determination of the structure of **2** as described below.

**Figure 4:**
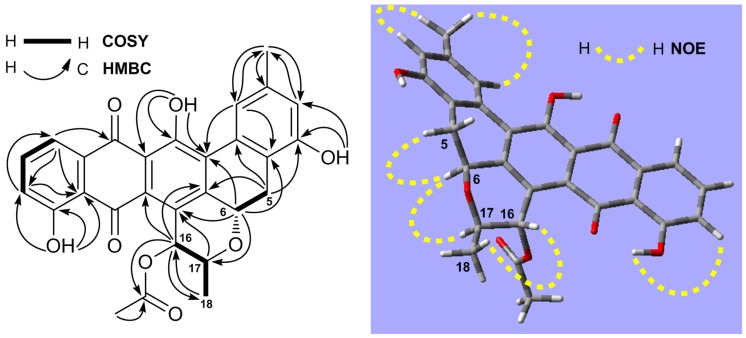
^1^H–^1^H COSY, key HMBC and NOE correlations of streptoanthraquinone A (**2**).

The ^1^H-NMR spectrum displayed five aromatic protons at δ 8.14 (d, 1.6 Hz), 7.01 (d, 1.6 Hz), 7.34 (dd, 7.5, 1.2 Hz), 7.71 (t, 7.5 Hz), and 7.81 (dd, 7.5, 1.2 Hz), which were assigned to H-1, H-3, H-10, H-11, and H-12, respectively. Three OH signals resonated at δ 12.84 (1H, s), 11.69 (1H, s), and 9.25 (1H, s), and the downfield shifts of δ 11.69 and 12.84 suggested that the protons from these two hydroxy groups had hydrogen bond relationships with carbonyls at C-8 and C-13. Further location of these three OH groups at C-4, C-9, and C-14 was made from the HMBC correlations, shown in Table 2 and Figure 4. One methyl (δ_C_ 22.6 and δ_H_ 2.50) was assigned at C-15 based on the HMBC correlations of H-15 (δ 2.50) with C-1 (δ 118.1), C-2 (δ 143.0), and C-3 (δ119.1), and both H-1 (δ 8.14) and H-3 (δ 7.01) with C-15 (δ 22.6). COSY correlations of H-5 with H-6 indicated their neighbor relationship of the methylene (δ_C_ 29.8 and δ_H_ 2.88, 3.57) at C-5 and the oxymethine at C-6 (δ_C_ 72.3 and δ_H_ 5.77). The HMBC correlations of H-5 with C-4 (δ 154.7), C-6a (δ 152.9), and C-14b (δ 118.8), and of H-6 with C-4a (δ 139.1), C-7 (δ 127.7), C-14a (δ 129.7), and C-17 (δ 77.1) demonstrated the presence of ring B. Similarly, the link sequence of the two oxymethines at C-16 (δ_C_ 73.1 and δ_H_ 5.33) and C-17 (δ_C_ 77.1 and δ_H_ 3.93) with the methyl at C-18 (δ_C_ 19.0 and δ_H_ 1.43, d, 6.1 Hz) was reduced from the COSY correlations of H-16 with H-17 and H-17 with H-18. The HMBC correlations of H-16 with C-6a, C-7a (δ 133.4), C-17, and C-18, and of H-17 with C-7, as well as a strong NOE correlation of H-17 with H-6, proved the presence of ring F. Finally, the acetyl group at C-16 was established by the HMBC correlations of H-16 with C-19 (δ 170.3) and of H-20 (δ 2.23) with C-19. The relative stereochemistry of **2** was proposed based on ^3^*J* coupling constants of protons and NOESY experiment. The ^3^*J* coupling constants of 12.1 Hz for αH-5/βH-6 and 9.5 Hz for αH-16/βH-17 indicated axial orientations for αH-5/βH-6 and αH-16/βH-17. NOESY spectrum showed strong NOE correlations of βH-6 (δ 5.77) with βH-5 (δ 3.57) and βH-17 (δ 3.93), and of αH-16 (δ 5.33) with αH-18 (δ 1.43), but no NOE for βH-6 with αH-5 (δ 2.88) or weak NOE for αH-16 with βH-17 were observed (Figure 4 and Figures S40). All of the above evidence suggested the orientations of βH-6, αH-16, and βH-17. The absolute configuration of **2** was proposed by a quantum chemical calculation of its ECD spectrum using the same method as that of **1**. Considering the rigid structure [27,28,29] of **2**, one preferred conformer (Figure S45) of (6*S*,16*R*,17*R*)-**2** was afforded by conformational analysis and geometrical optimization. The ECD spectrum was then calculated using the TDDFT method. As shown in Figure 3B, the experimental ECD curve of **2** was similar to the calculated ECD spectrum of (6*S*,16*R*,17*R*)-**2** rather than of (6*R*,16*S*,17*S*)-**2**. Thus, the absolute configuration of **2** was proposed to be 6*S*, 16*R*, 17*R*. The full ^1^H and ^13^C assignments (Table 2) of **2** were achieved by extensive NMR spectral analysis. Compound **2** was elucidated as streptoanthraquinone A, a novel dihydrobenzo[*a*]naphthacenequinone [26] with a rare oxygen-contained hexatomic ring F at C-6 and C-7 positions. The skeleton of dihydrobenzo[*a*]naphthacenequinone, such as benaphthamycin [30], benanomicins, ericamycin, pradimicins, and WS-79089A [26], usually has five rings, A, B, C, D, and E. Benaphthamycin, ericamycin, and WS-79089A have an additional ring F at C-2 and C-3 positions, while compound **2** had its sixth ring F at C-6 and C-7 positions, which is unique. To the best of our knowledge, such a structure is the first that has ever been found in the class of dihydrobenzo[*a*]naphthacenequinones.

**Table 2:** ^13^C- and ^1^H-NMR Data of streptoanthraquinone A (**2**) (in CDCl_3_, *J* = Hz).

No.	δ_C_, Type	δ_H_ (*J* = Hz)	HMBC	No.	Δ_c_, Type	δ_H_ (*J* = Hz)	HMBC
1	118.1, CH	8.14, d (1.6)	3, 4a, 14a, 14b, 15	12a	135.3, C		
2	143.0, C			13	189.6, C		
3	119.1, CH	7.01, d (1.6)	1, 4, 4a, 15	13a	118.9 ^a^, C		
4	154.7, C			14	154.3, C		
4a	139.1, C			14a	129.7, C		
5	29.8, CH_2_	2.88, dd (15.6, 12.1); 3.57, dd (15.6, 3.3)	4, 6, 6a 6, 6a, 14b	14b	118.8 ^a^, C		
6	72.3, CH	5.77, dd (12.1, 3.3)	4a, 7, 14a, 17	15	22.6, CH_3_	2.50, s	1, 2, 3
6a	152.9, C			16	73.1, CH	5.33, d (9.5)	6a, 7a, 17, 18, 19
7	127.7, C			17	77.1, CH	3.93, dd (9.5, 6.1)	7
7a	133.4, C			18	19.0, CH_3_	1.43, d (6.1)	16, 17
8	193.5, C			19	170.3, C		
8a	114.9, C			20	21.0, CH_3_	2.23, s	19
9	162.2, C			4-OH		9.25, s	3, 4
10	125.5, CH	7.34, dd (7.5, 1.2)	8a, 9, 12	9-OH		11.69, s	8a, 9, 10
11	138.1, CH	7.71, t (7.5)	9, 12a	14-OH		12.84, s	13a, 14, 14a
12	121.7, CH	7.81, dd (7.5, 1.2)	8a, 10, 13				

^a^ The data with the same label in each column may be interchanged.

### 2.2. Biological Activities

**Figure 5:**
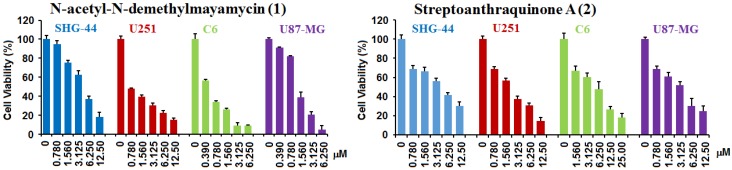
*N*-acetyl-*N*-demethylmayamycin (**1**) and streptoanthraquinone A (**2**) inhibited the proliferation of glioma SHG-44, U251, C6, and U87-MG cells. Glioma cells were treated with **1** or **2** for 72 h at different concentrations. Values are means ± S.D. from five independent experiments.

The compounds **1** and **2** were assayed for their activities inhibiting the proliferation of glioma C6, U251, U87-MG, and SHG-44 cells using the sulforhodamine B (SRB) assay. The SRB assay is a method that measures total cellular protein content for evaluating the activity of tested compounds against the proliferation of tumor cells. Doxorubicin (DOX) was used as a positive control. Glioma cells were treated with tested compounds for 72 h at different concentrations. The results (Table 3 and Figure 5) showed that *N*-acetyl-*N*-demethylmayamycin (**1**) and streptoanthraquinone A (**2**) significantly inhibited the proliferation of four different tested glioma cell lines with IC_50_ values in a range from 0.5–3.9 μM for **1** and 3.3–7.3 μM for **2**. The control DOX displayed activities with IC_50_ 0.9–9.0 μM. *N*-acetyl-*N*-demethylmayamycin (**1**) and streptoanthraquinone A (**2**) were also assayed for their activity against normal human astrocytes (HA). The initial assay was conducted on the concentrations from 0.35 to 7.0 μM for **1** and on those from 1.65 to 16.5 μM for **2**. The cell viability of HA cells from each tested concentration of both compounds was 100%. The results from the second assay in higher concentrations showed (Table 3) that the IC_50_ values of compounds **1** and **2** against HA cells were 25 μM for **1** and >100 μM for **2**. The ratios of IC_50_ for human astrocytes (IC_50HA_) to IC_50_ for glioma cells (IC_50gc_) were 6.4–53 for **1** and >14–31 for **2**.

**Table 3:** Activity of *N*-acetyl-*N*-demethylmayamycin (**1**) and streptoanthraquinone A (**2**) inhibiting the proliferation of cells (IC_50_: μM).

Compounds	U251	U87-MG	SHG-44	C6	HA
*N*-acetyl-*N*-demethylmayamycin (**1**)	0.7 ± 0.2	1.4 ± 0.1	3.9 ± 0.4	0.5 ± 0.1	25 ± 1.3
Ratios of IC_50HA_/IC_50gc_	35	18	6.4	53	
Streptoanthraquinone A (**2**)	3.3 ± 0.3	4.6 ± 0.3	6.5 ± 1.1	7.3 ± 1.4	>100
Ratios of IC_50HA_/IC_50gc_	>31	>22	>16	>14	
Doxorubicin (DOX)	6.7 ± 1.1	0.9 ± 0.1	9.0 ± 0.8	1.0 ± 0.1	NT

NT: No testing.

**Figure 6:**
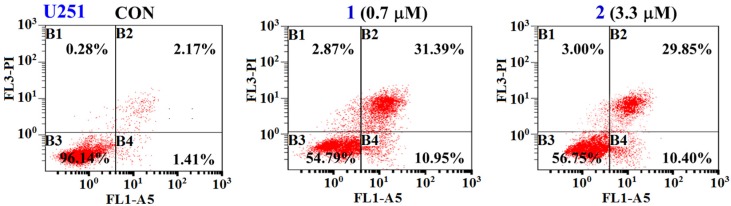
*N*-acetyl-*N*-demethylmayamycin (**1**) and streptoanthraquinone A (**2**) induced apoptosis in the glioma U251 cells quantified by cytometric analysis. U251 cells were treated with **1** (0.7 μM) or **2** (3.3 μM) for 36 h and then stained with Annexin-V FITC and PI double staining (B1: necrotic cells; B2: late apoptotic cells; B3: normal glioma cells; B4: early apoptotic cells).

*N*-acetyl-*N*-demethylmayamycin (**1**) and streptoanthraquinone A (**2**) were further tested for their ability to induce apoptosis in glioma U251 cells by flow cytometry using Annexin V-FITC/PI double staining. U251 cells were treated with *N*-acetyl-*N*-demethylmayamycin (**1**) and streptoanthraquinone A (**2**) in their IC_50_ concentrations of 0.7 μM for **1** and 3.3 μM for **2** for 36 h, stained with annexin-V FITC and PI, and then analyzed by using flow cytometry. It was found that the total apoptotic cells including early and late apoptotic cells were increased by 38.76% for **1** and 36.67% for **2** when compared to the control (CON, 3.58%, Figure 6). These data demonstrated that *N*-acetyl-*N*-demethylmayamycin (**1**) and streptoanthraquinone A (**2**) significantly induced apoptosis in the glioma U251 cells.

Compounds **1** and **2** were also determined for their activities against methicillin-resistant *Staphylococcus aureus* ATCC 43300 and *Escherichia coli* ATCC 25922. The result indicated that only *N*-acetyl-*N*-demethylmayamycin (**1**) inhibited the growth of *S. aureus* with MIC 20.0 μM. The positive control norfloxacin inhibited the growth of both *S. aureus* and *E. coli* with MIC values of 62.6 μM and 31.3 μM, respectively. However, both compounds showed no activity against *E. coli*.

## 3. Experimental Section 

### 3.1. General Experimental Procedures

Optical rotations were measured on a JASCO DIP-370 digital polarimeter. CD spectra were recorded on a JASCO J 715 spectropolarimeter. IR spectra were recorded on an AVATAR 370 FT-IR spectrometer (Thermo Nicolet, Madison, WI, USA). NMR spectra were acquired on a Bruker 500 spectrometer using standard pulse programs and acquisition parameters. Chemical shifts were expressed in δ (ppm) and referred to the NMR solvent used. HRESIMS data were acquired on an Agilent 6230 TOF LC/MS spectrometer. Octadecyl-functionalized silica gel (ODS, Cosmosil 75C18-Prep, Nacalai Tesque Inc., Kyoto, Japan) was used for column chromatography. HPLC purification was performed on an Agilent 1260 HPLC system with DAD detector. HPLC and analytic grade solvents used for this study were purchased from the Sinopharm Chemical Reagent Co. Ltd. (Shanghai, China). Human glioma U251, U87-MG, and SHG-44 cells, and rat glioma C6 cells, were obtained from the Cell Bank of the Chinese Academy of Sciences. Normal human astrocytes (HA, Cat. No. 1800) were obtained from ScienCell. The methicillin-resistant *Staphylococcus aureus* ATCC 43300 and *Escherichia coli* ATCC 25922 were gifts from Professor Zhongjun Ma and Dr. Pinmei Wang. Nutrient Broth (NB), Mueller Hinton Broth (MHB), and Gause’s-agar were purchased from Hangzhou Microbial Reagent Co. Ltd. (Hangzhou, China), Thermo Fisher Scientific Inc. (Waltham, MA, USA), and Guangdong Huankai Microbial Science and Technology Co. Ltd. (Guangzhou, China), respectively. Doxorubicin (DOX, >98.0%) was obtained from Sigma-Aldrich and norfloxacin (98%) from Saen Chemical Technology Co. Ltd. (Shanghai, China).

### 3.2. Isolation and Taxonomic Identity of Marine Streptomyces sp. 182SMLY

Strain 182SMLY was derived from a sample of marine sediment at a 3.6 m depth, which was collected from the East China Sea, close to Zhoushan City, Zhejiang Province, China in August 2013. Briefly, the marine sediments (3.0 g) were air-dried for five days in a sterile centrifuge tube. The dried sample was diluted into 0.01 g/mL with seed broth (1.5% glucose, 1.5% glycerol, 1.5% malt extract, 2.5% yeast extract, 0.5% casamino acids, and 0.1% calcium carbonate). The diluted sample (200 μL) was dispersed across a Bacto-agar plate and then incubated at room temperature for 10 days. Bactria colonies were picked with sterile needles and transferred to Bacto-agar plates. After another seven days of growth at room temperature, the single colony (strain 182SMLY) that grew well was transferred onto Gause’s synthetic agar media. Working stocks were prepared on Gause’s synthetic agar slants and stored at 4 °C until use.

16S rDNA analysis was used to determine the taxonomic identity of strain 182SMLY, and the DNA sequence using BLAST (nucleotide sequence comparison) was compared to the GenBank database. The 16S rDNA sequence of strain 182SMLY has been deposited in GenBank (accession number: KT899860). A voucher strain (*Streptomyces* sp. 182SMLY) of this actinomycete was preserved at the Laboratory of Institute of Marine Biology, Ocean College, Zhejiang University, China.

### 3.3. Culture of Strain Streptomyces sp. 182SMLY

Colonies of the strain growing on Gause’s synthetic agar media were inoculated into a 500 mL Erlenmeyer flask containing 200 mL of a liquid medium (20 g/L starch, 1.0 g/L KNO_3_, 0.5 g/L K_2_HPO_4_, 0.5 g/L MgSO_4_·7H_2_O, 0.5 g/L NaCl, 0.01 g/L FeSO_4_·7H_2_O), and the colonies were then incubated at 28 °C for 5 days on a rotary shaker (180 rpm) to produce seed broth. The seed broth (5 mL) was then inoculated into a 500 mL Erlenmeyer flask that contained 250 mL of liquid Gause’s synthetic media. The flask was incubated at 28 °C for 10 days on a rotary shaker (180 rpm). A total of 30 L fermentation was made for this study.

### 3.4. Extraction and Isolation of Compounds

The fermentation broth (30 L) of the isolated marine *Streptomyces* sp. 182SMLY was filtered with a filter press to give filtrate and mycelia. The filtrate was extracted with EtOAc three times (each 10.0 L), and the mycelia were extracted with MeOH. The EtOAc and MeOH phases were combined and then dried *in vacuo* to afford a crude extract (5.98 g). This crude extract was fractionated by column chromatography of ODS (10 × 150 cm) successively eluting with 70%, 85%, and 100% MeOH to yield three fractions (Frs. 1–3). Fr. 3 was further separated by HPLC using an Agilent column (Zorbax SB-C18, 250 × 9.4 mm, 5 μm) with an isocratic mobile phase of MeOH and H_2_O (80:15) at a flow rate of 1.0 mL/min and UV detection wavelength of 256 nm to give *N*-acetyl-*N*-demethylmayamycin (**1**, 4.5 mg, *t*_R_ 16.46 min) and streptoanthraquinone A (**2**, 2.3mg, *t*_R_ 21.45 min).

*N-acetyl-N-demethylmayamycin (**1**)*: dark brown amorphous powder; molecular formula C_27_H_25_NO_8_; *t*_R_ 16.48 min (85% MeOH in H_2_O); ${\left[\alpha \right]}_{D}^{25}$ + 63.67 (*c* 0.50, DMSO); UV (MeOH) λ_max_ (log ε) 217 (4.70), 236 (4.69), 328 (4.36), 443 (4.06) nm; ECD (10 mg/L, MeOH) λ_max_ (Δε) 212 (−38.1), 238 (+4.0), and 325 (−14.8) nm; IR (KBr) ν_max_ 3426, 2925, 2853, 1732, 1712, 1629, 1598, 1458, 1375, 1066, 1024 cm^−1^; ^1^H (500 MHz, in DMSO-*d*_6_) and ^13^C (125 MHz, in DMSO-*d*_6_) NMR data, see Table 1; HRESIMS *m/z* [M + Na]^+^ 514.1457 (calcd for C_27_H_25_NNaO_8_, 514.1478).

*Streptoanthraquinone A (**2**):* dark brown amorphous powder; molecular formula C_28_H_22_O_8_; *t*_R_ 21.45 min (85% MeOH in H_2_O); ${\left[\alpha \right]}_{D}^{25}$ + 51.90 (*c* 0.50, DMSO); UV (MeOH) λ_max_ (log ε) 220 (4.70), 330 (3.40), 445 (3.06) nm; ECD (10 mg/L, MeOH) λ_max_ (Δε) 215 (−59.2), 236 (+39.1, and 325 (−20.0) nm; IR (KBr) ν_max_ 3435, 2925, 2853, 1738, 1712, 1639, 1459, 1381, 1024 cm^−1^; ^1^H (500 MHz, in DMSO-*d*_6_) and ^13^C (125 MHz, in DMSO-*d*_6_) NMR data, see Table 2; HRESIMS *m/z* [M + Na]^+^ 509.1233 (calcd for C_28_H_22_NaO_8_, 509.1212).

### 3.5. Computational Methods

Conformational analysis was performed with a MacroModel employing an OPLS force field. Geometrical optimization and energy calculations were performed applying the DFT method at the B3LYP/6-31+G(d) level with the Gaussian 09 program package [31]. The TDDFT calculation was run at the same level and the ECD spectra were generated by the program SpecDis v1.53 and were summed according to the Boltzmann-weighting of each individual conformer.

### 3.6. Cells Culture

Human glioma U251 and rat glioma C6 cells were cultured in DMEM (Dulbecco’s Modified Eagle Medium, Gibco, Thermo Fisher Scientific Inc., Waltham, MA, USA) with 10% FBS (Fetal Bovine Serum, PAA Laboratories Inc., Dartmouth, MA, USA), human glioma SHG-44 in RPMI-1640 Medium (Roswell Park Memorial Institution 1640 Medium, Gibco, Thermo Fisher Scientific Inc., Waltham, MA, USA), human glioma U87-MG cells in MEM (Minimum Essential Medium, Gibco), and normal human astrocytes (HA) in AM (Astrocyte Medium, ScienCell, Cat. No. 1801). All cells were incubated at 37 °C in a humidified incubator with 5% CO_2_. Cells after the third generation were used for experiment.

### 3.7. Sulforhodamine B (SRB) Assay

The SRB assay [8,9] was used to evaluate the activity of the isolated compounds in inhibiting the proliferation of glioma U87-MG, U251, SHG-44, and C6 cells. Doxorubicin (DOX) was used as the positive control. Briefly, glioma cells were plated in a 96-well plate and then treated with different concentrations of tested compound after cells adhesion for 24 h. After 72 h of the treatment, cells were fixed with 50 μL of a 10% cold TCA solution for 1 h at 4 °C, washed with distilled water five times, and then dried at room temperature. The dried cells were stained with 50 μL of 0.4% SRB for ten minutes and rinsed with a 1% acetic acid solution five times. After dried, dye was dissolved in a 10 mM Tris buffer and measured at 515 nm on a microplate reader (BioTech, Winooski, VT, USA).

### 3.8. Antimicrobial Assay

The micro broth dilution method as described in the previous report [12] was used to determine the antimicrobial activity of the isolated compounds against the methicillin-resistant *Staphylococcus aureus* ATCC 43300 and *Escherichia coli* ATCC 25922. Norfloxacin, a broad-spectrum antibiotic against both Gram-positive and Gram-negative bacteria, was used as the positive control.

## 4. Conclusions 

Two new anthraquinones were isolated from the culture of *Streptomyces* sp. 182SMLY, which was isolated from a marine sediment sample. Their structures were determined to be *N*-acetyl-*N*-demethylmayamycin (**1**) and streptoanthraquinone A (**2**) based on the extensive spectroscopic analysis including 2D NMR, HRESIMS and the electronic circular dichroism (ECD) calculation. Both new compounds significantly inhibited the proliferation of four different glioma cell lines with IC_50_ values of 0.5 to 7.3 μM and induced apoptosis in the glioma cells. *N*-acetyl-*N*-demethylmayamycin (**1**) also showed strong activity against the growth of methicillin-resistant *Staphylococcus aureus* with MIC 20.0 μM.

## Supplementary Materials

The following are available online at www.mdpi.com/1660-3397/14/1/10. UV, NMR, and HRESIMS spectra of compounds **1** and **2** as well as other supporting data.

## Abbreviations

DFT: Density functional theory
DOX: Doxorubicin
ECD: Electronic circular dichroism
ODS: Octadecyl-functionalized silica gel
OPLS: Optimized potentials for liquid simulations
SRB: Sulforhodamine B
TDDFT: Time-dependent density functional theory
TMZ: Temozolomide
