# Supplementary material for: Bioactive Polycyclic Quinones from Marine Streptomyces sp. 182SMLY
Source: Mar Drugs. 2016 Jan 6;14(1):10. doi: 10.3390/md14010010 (PMC4728507; doi:10.3390/md14010010)
Supplement: Supplementary file 1 [file marinedrugs-14-00010-s001.pdf]

# Supplementary Materials: Bioactive Polycyclic Quinones from Marine *Streptomyces* sp. 182SMLY

**Ying Liang, Xin Xie, Lu Chen, Shilun Yan, Xuewei Ye, Komal Anjum, Haocai Huang, Xiao-Yuan Lian and Zhizhen Zhang**

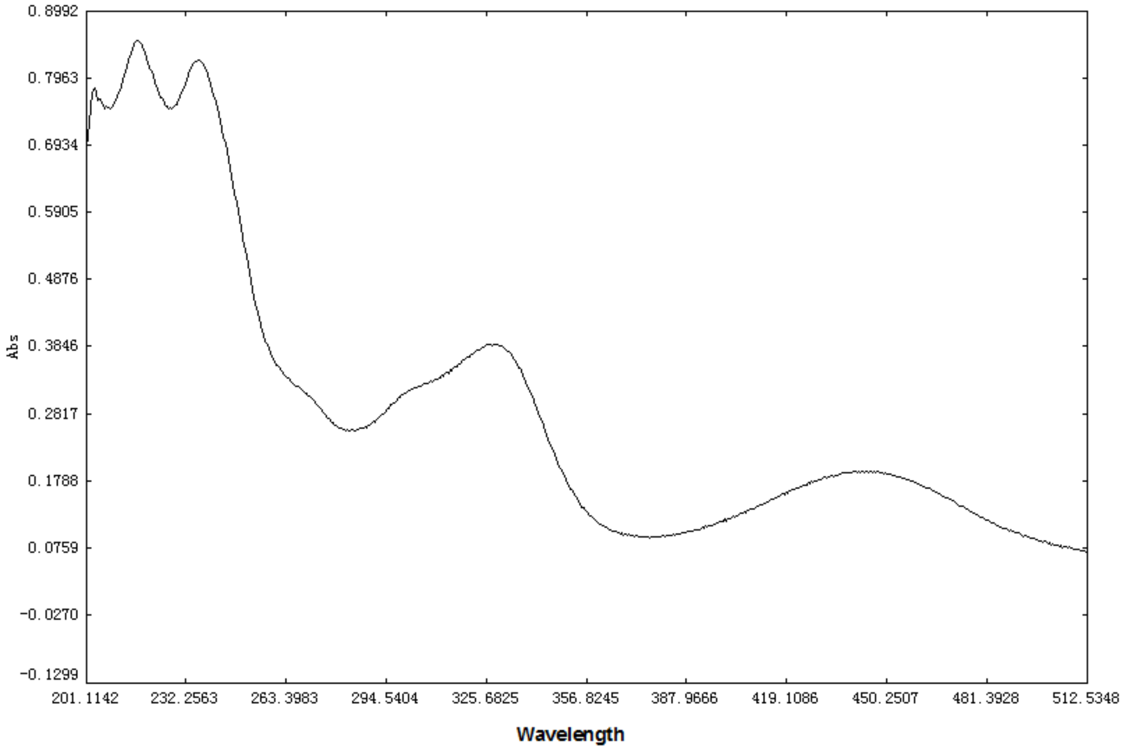


**Figure S1.** UV Spectrum of *N*-acetyl-*N*-demethylmayamycin (**1**).

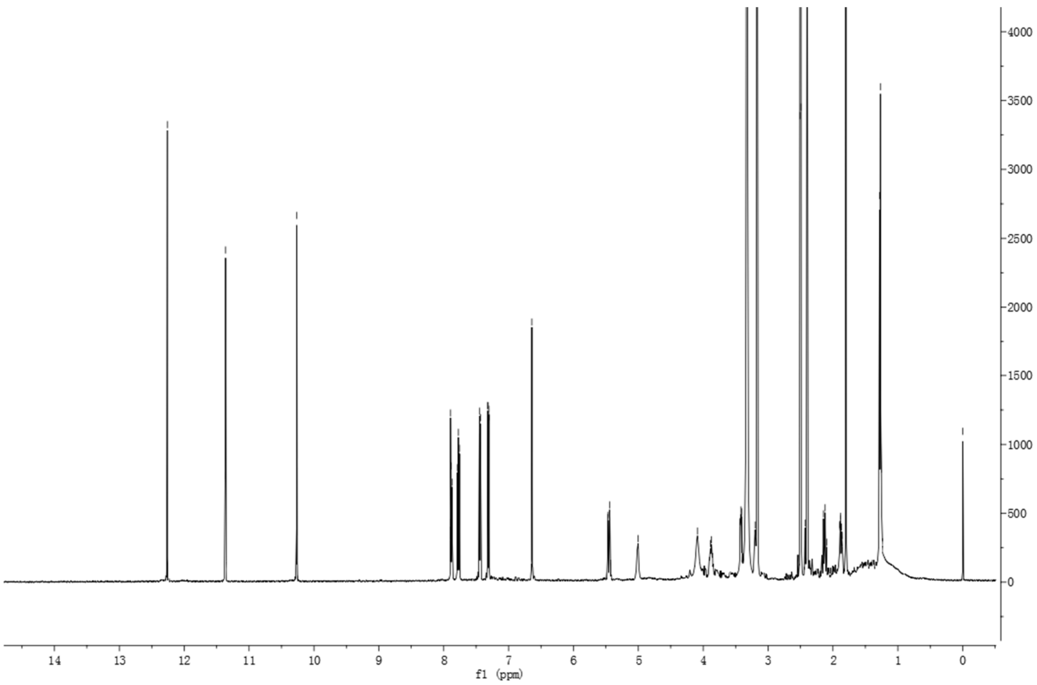


**Figure S2.** 1H NMR Spectrum of *N*-acetyl-*N*-demethylmayamycin (**1**).

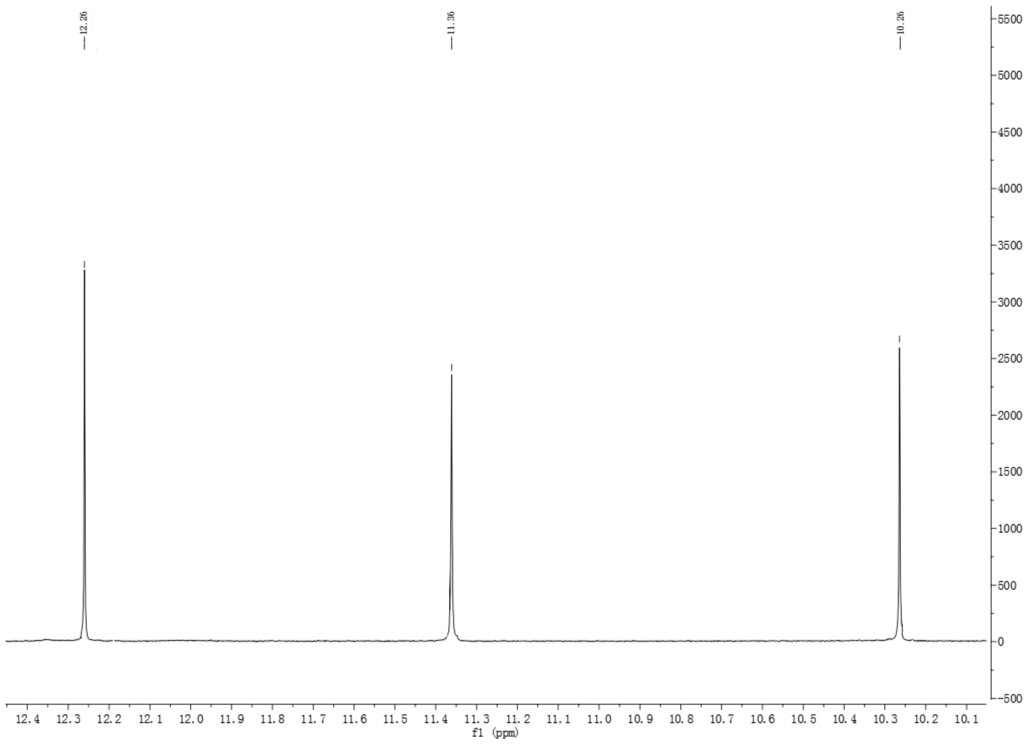


**Figure S3.** 1H NMR Spectrum of *N*-acetyl-*N*-demethylmayamycin (**1**).

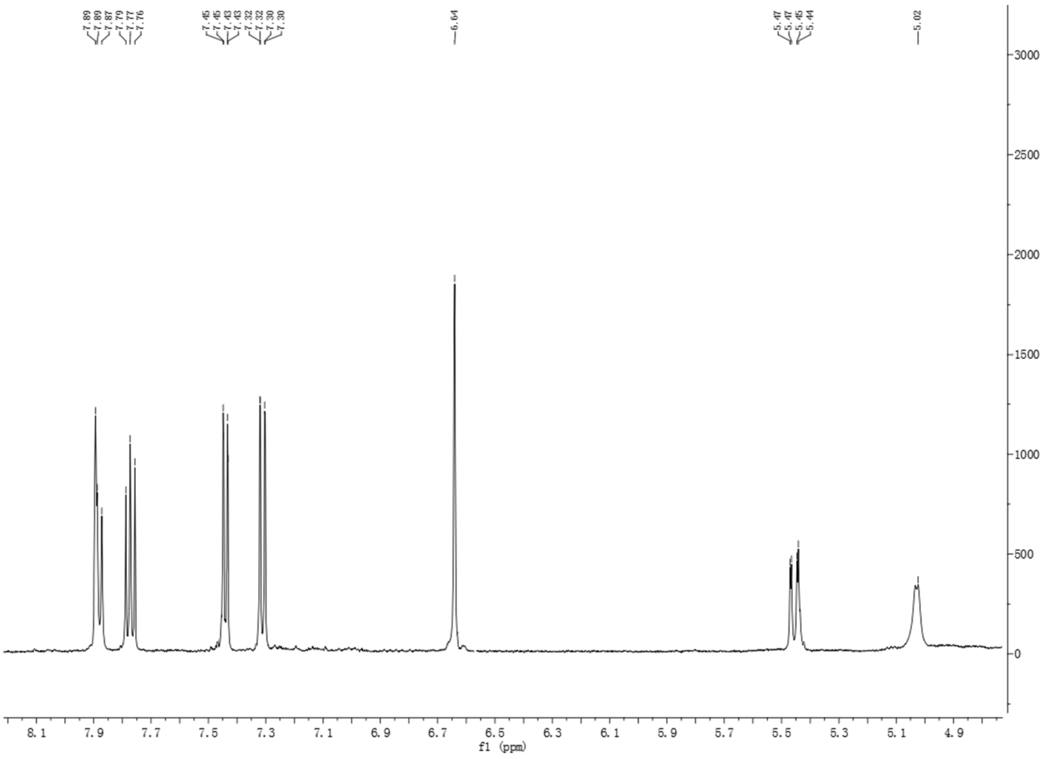


**Figure S4.** 1H NMR Spectrum of *N*-acetyl-*N*-demethylmayamycin (**1**).

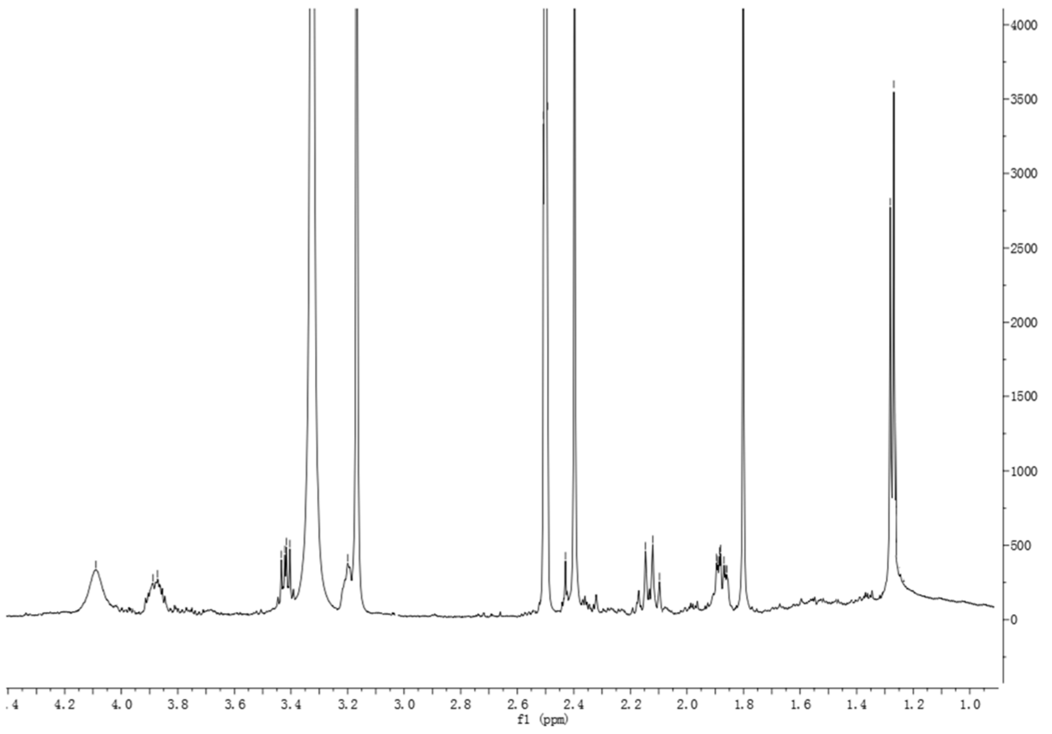


**Figure S5.** 1H NMR Spectrum of *N*-acetyl-*N*-demethylmayamycin (**1**).

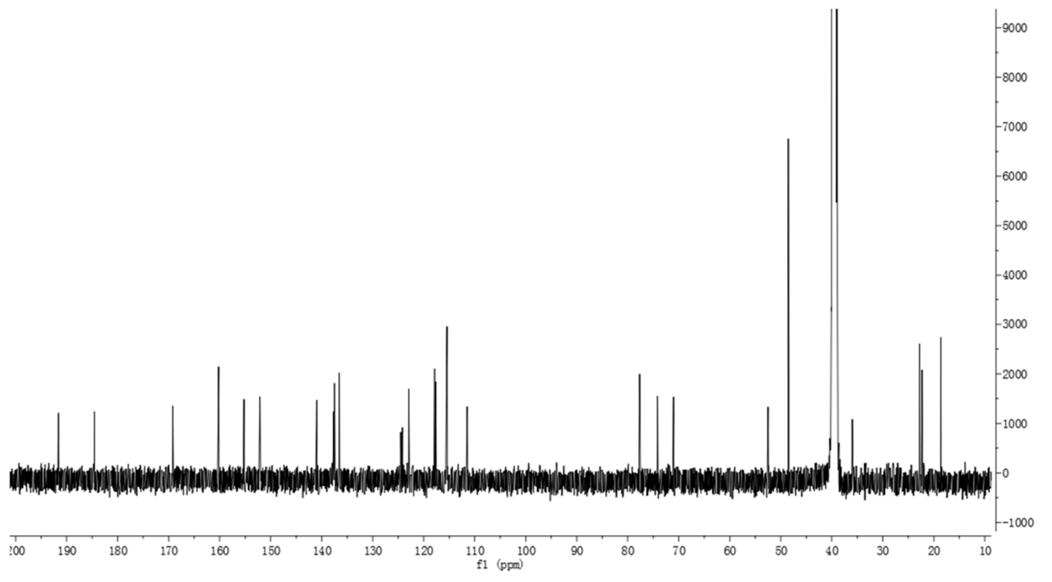


**Figure S6.** 13C-NMR of *N*-acetyl-*N*-demethylmayamycin (**1**).

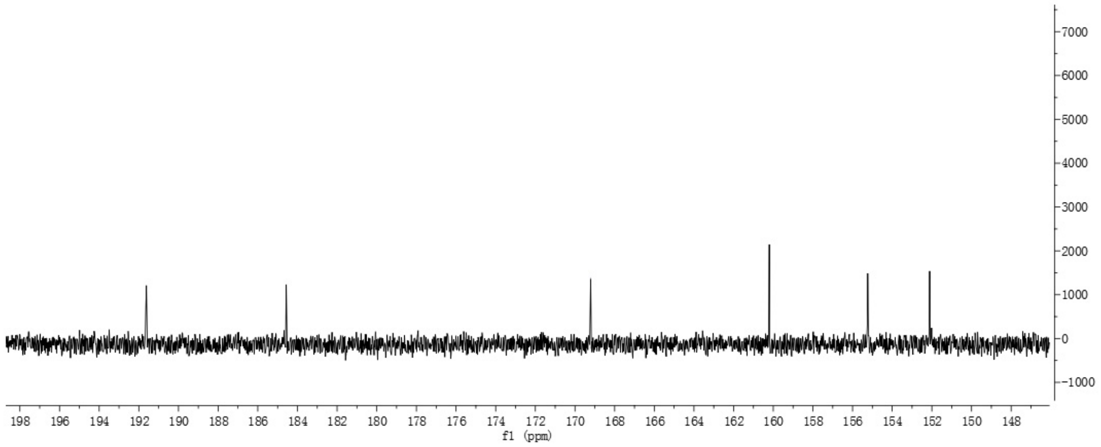


**Figure S7.** 13C-NMR of *N*-acetyl-*N*-demethylmayamycin (**1**).

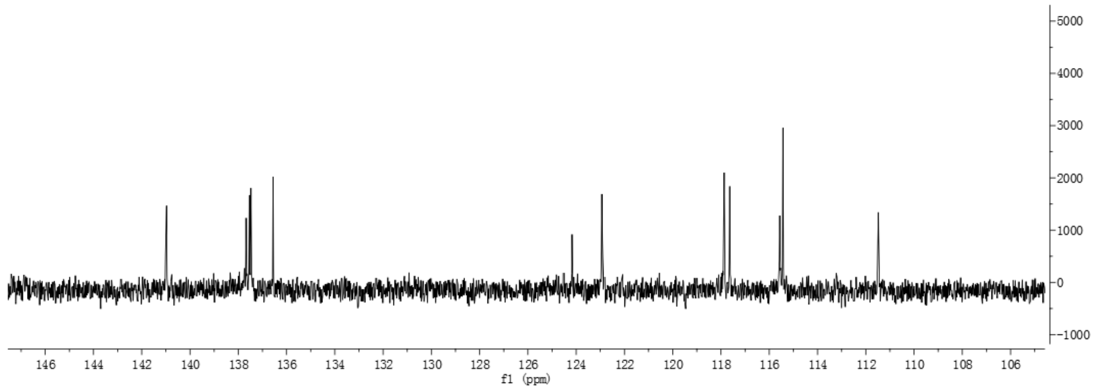


**Figure S8.** 13C-NMR of *N*-acetyl-*N*-demethylmayamycin (**1**).

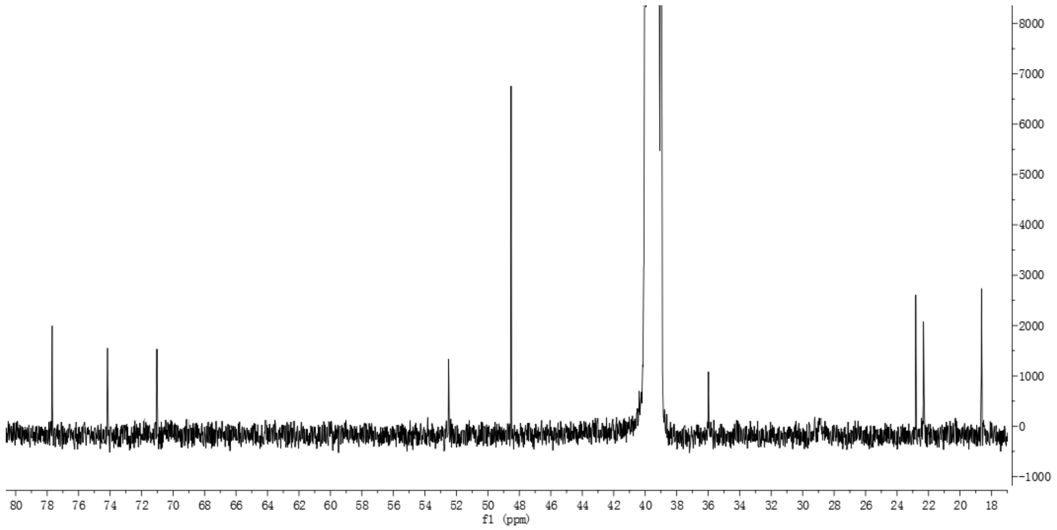


**Figure S9.** 13C-NMR of *N*-acetyl-*N*-demethylmayamycin (**1**).

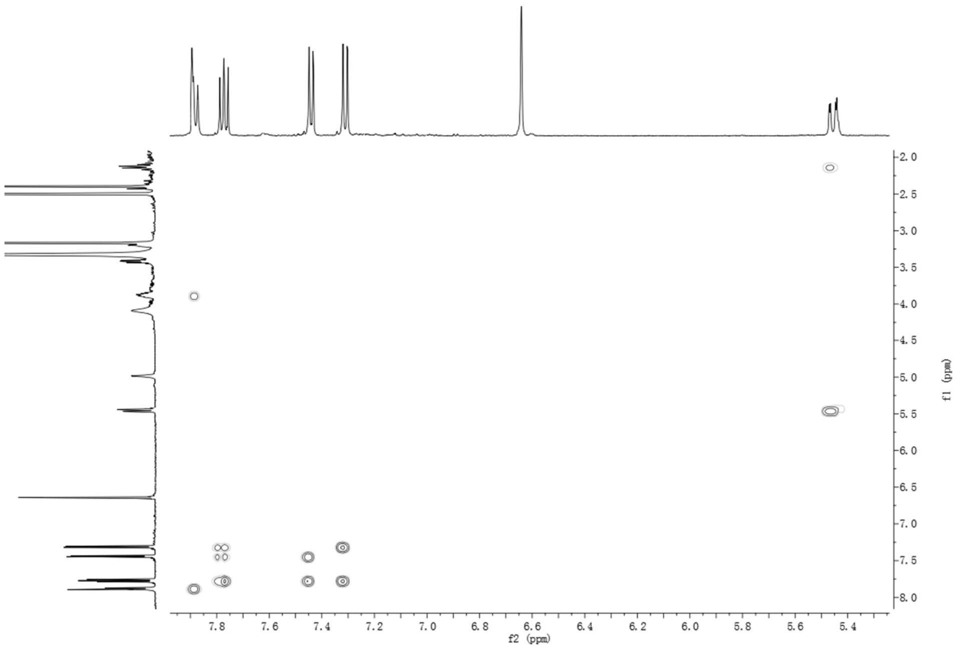


**Figure S10.** 1H-1H COSY Spectrum of *N*-acetyl-*N*-demethylmayamycin (**1**).

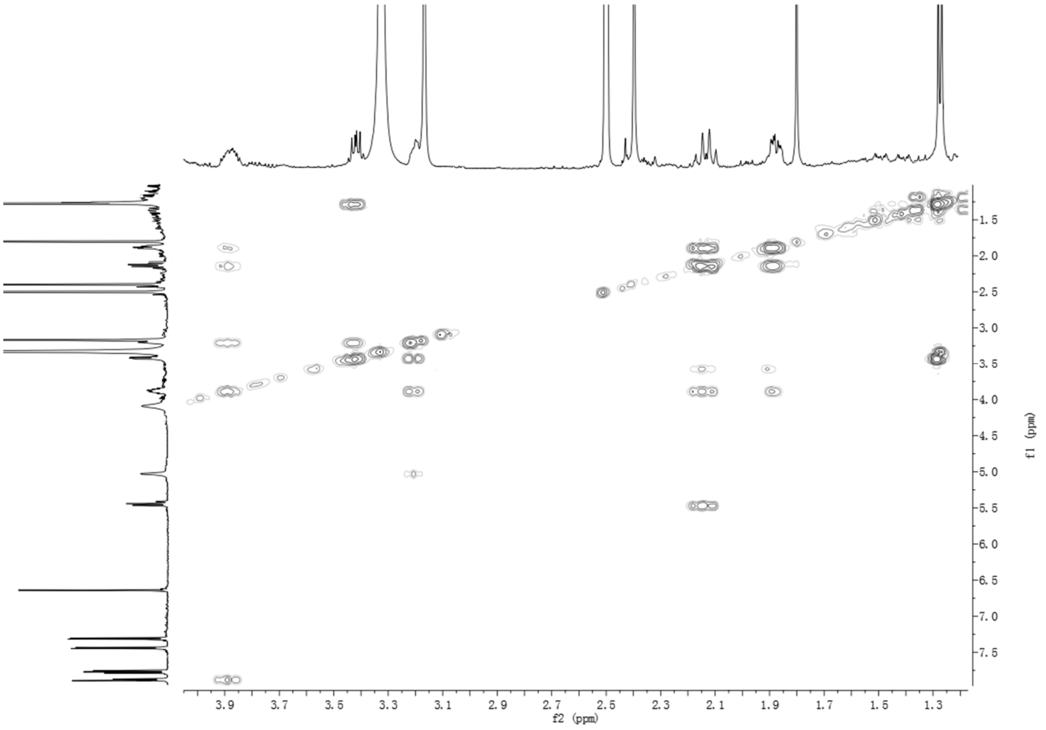


**Figure S11.** 1H-1H COSY Spectrum of *N*-acetyl-*N*-demethylmayamycin (**1**).

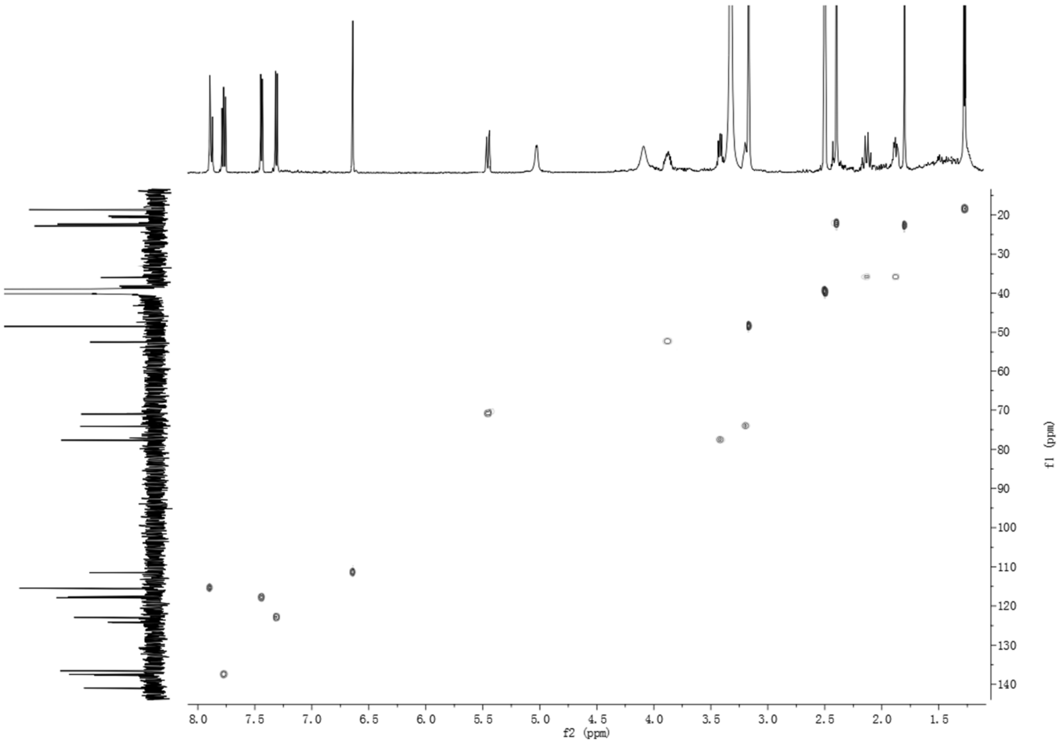


**Figure S12.** HSQC Spectrum of *N*-acetyl-*N*-demethylmayamycin (**1**).

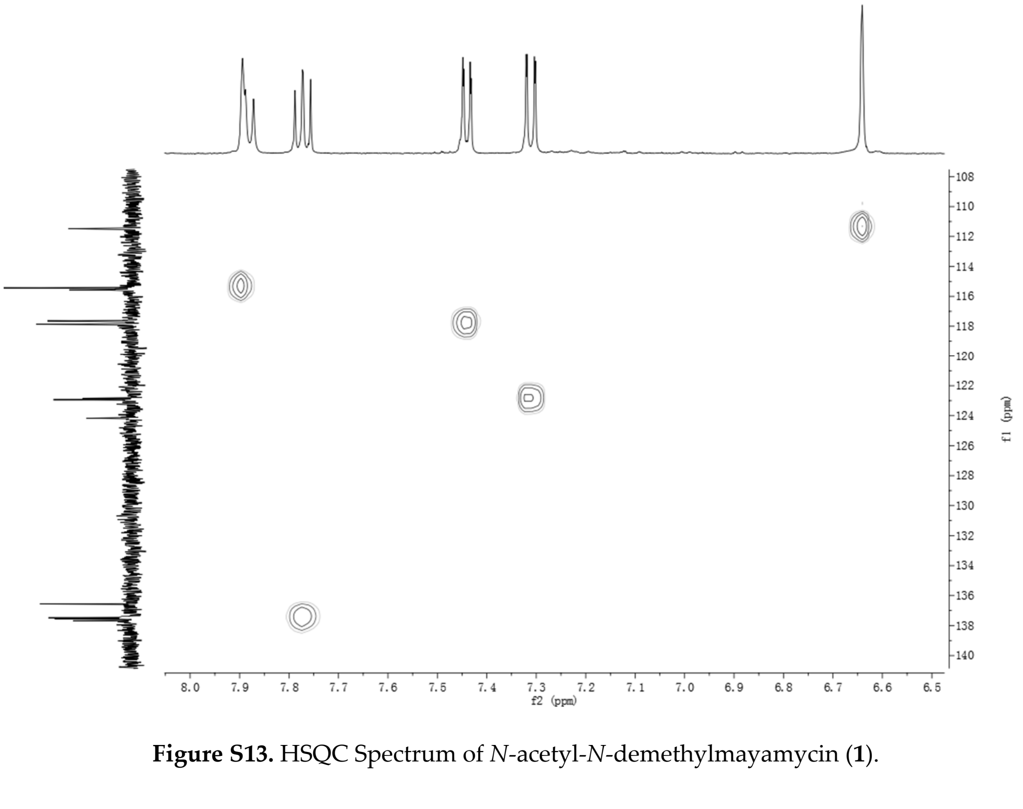


**Figure S13.** HSQC Spectrum of *N*-acetyl-*N*-demethylmayamycin (**1**).

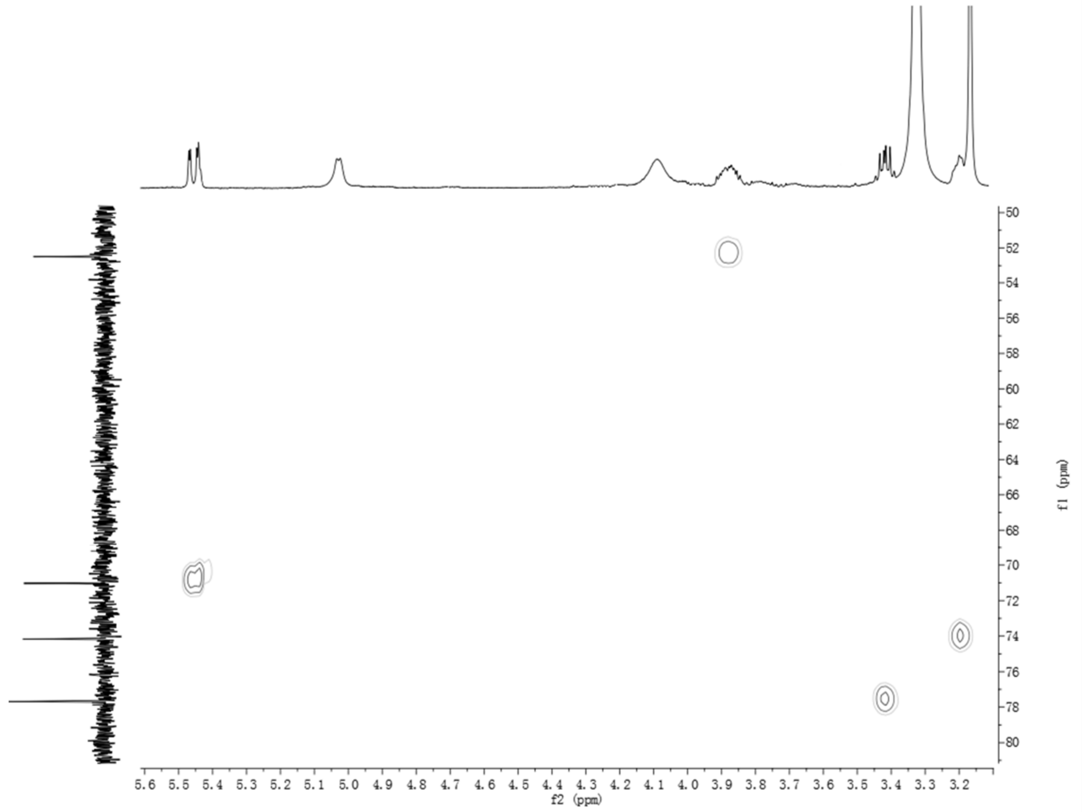


**Figure S14.** HSQC Spectrum of *N*-acetyl-*N*-demethylmayamycin (**1**).

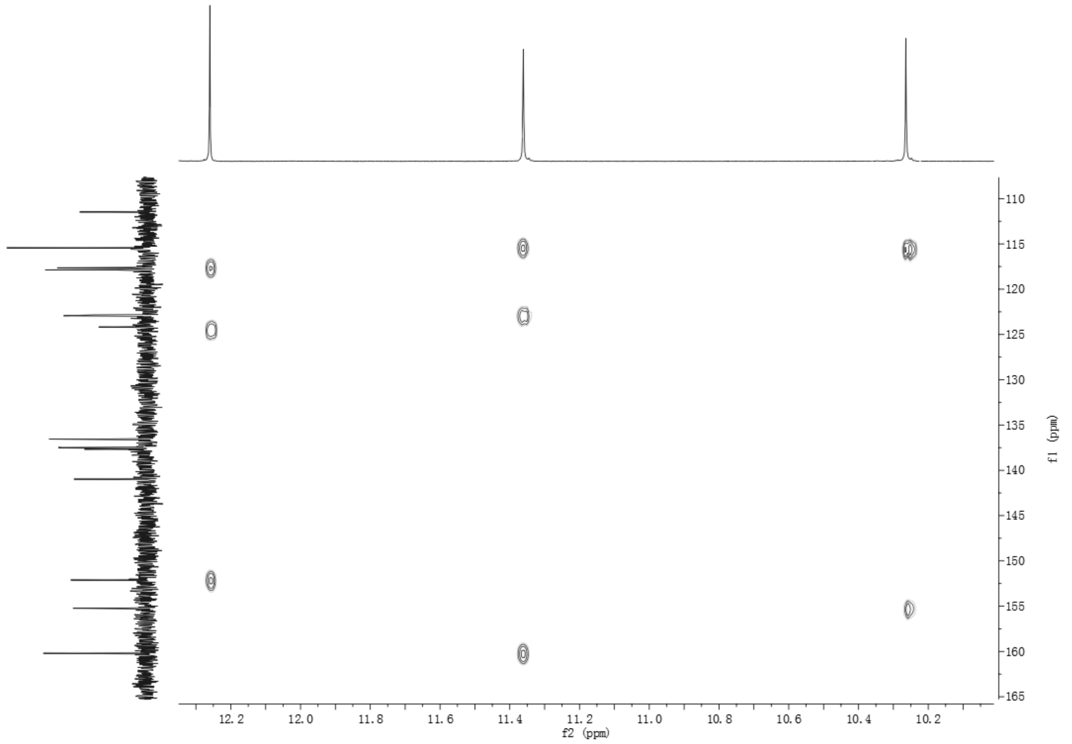


**Figure S15.** HMBC Spectrum of *N*-acetyl-*N*-demethylmayamycin (**1**).

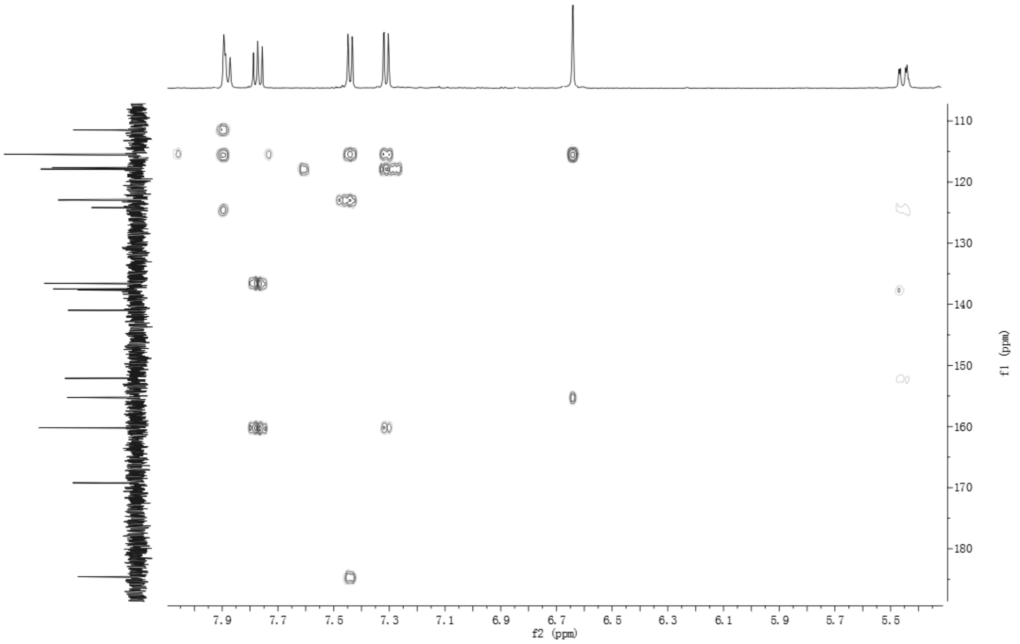


**Figure S16.** HMBC Spectrum of *N*-acetyl-*N*-demethylmayamycin (**1**).

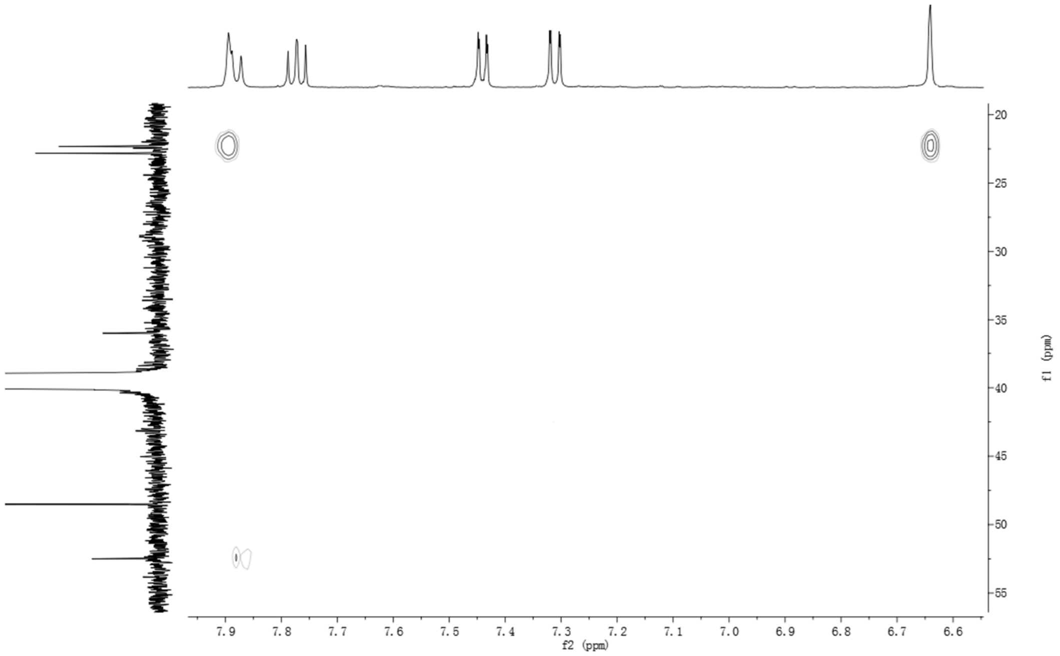


**Figure S17.** HMBC Spectrum of *N*-acetyl-*N*-demethylmayamycin (**1**).

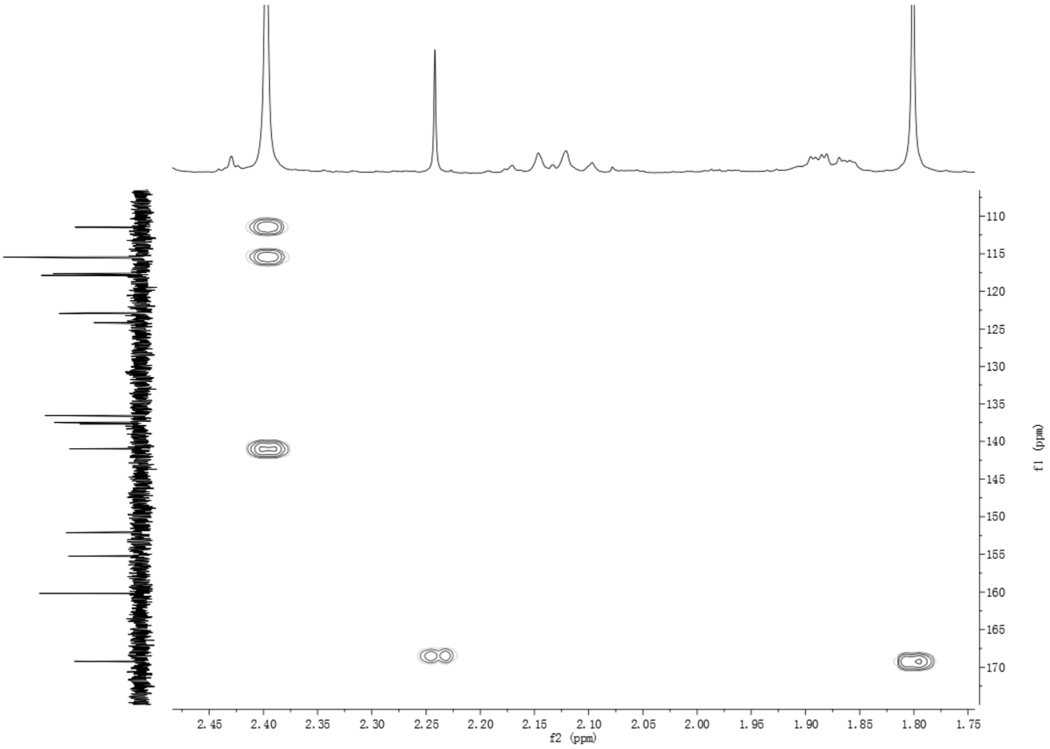


**Figure S18.** HMBC Spectrum of *N*-acetyl-*N*-demethylmayamycin (**1**).

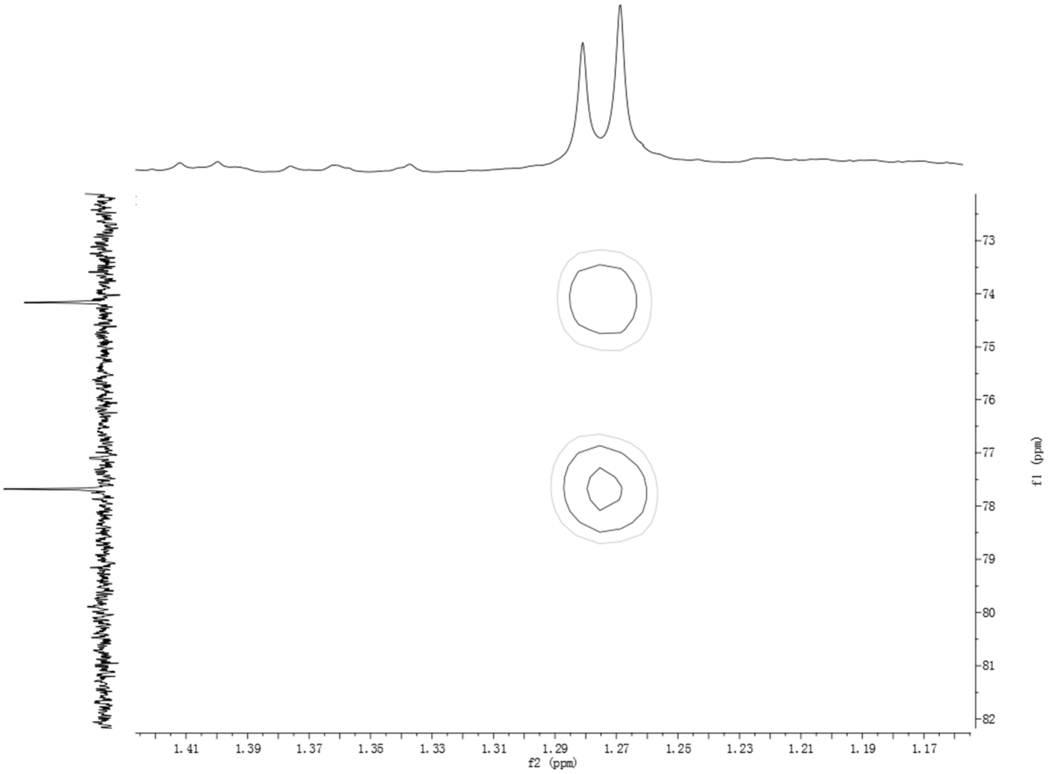


**Figure S19.** HMBC Spectrum of *N*-acetyl-*N*-demethylmayamycin (**1**).

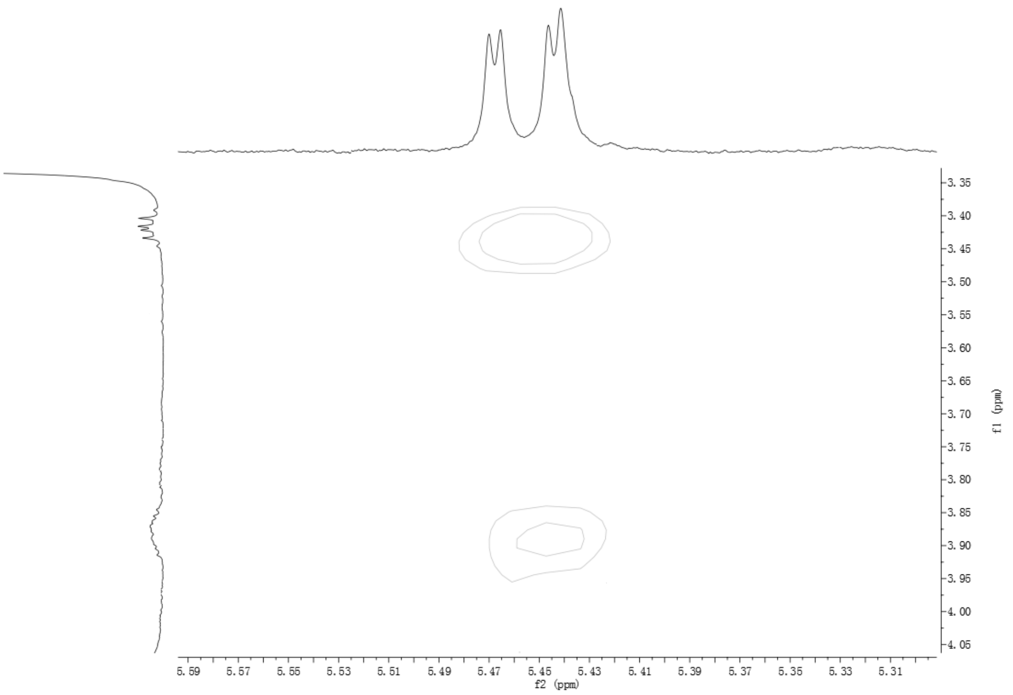


**Figure S20.** NOESY Spectrum of *N*-acetyl-*N*-demethylmayamycin (**1**).

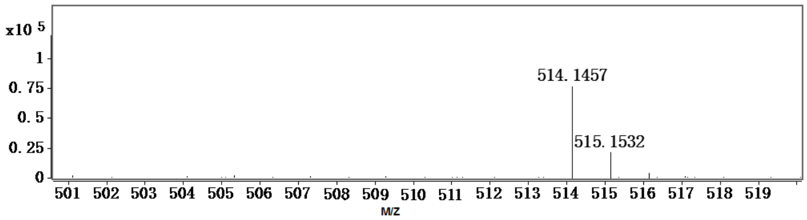


**Figure S21.** HRESIMS Data of *N*-acetyl-*N*-demethylmayamycin (**1**).

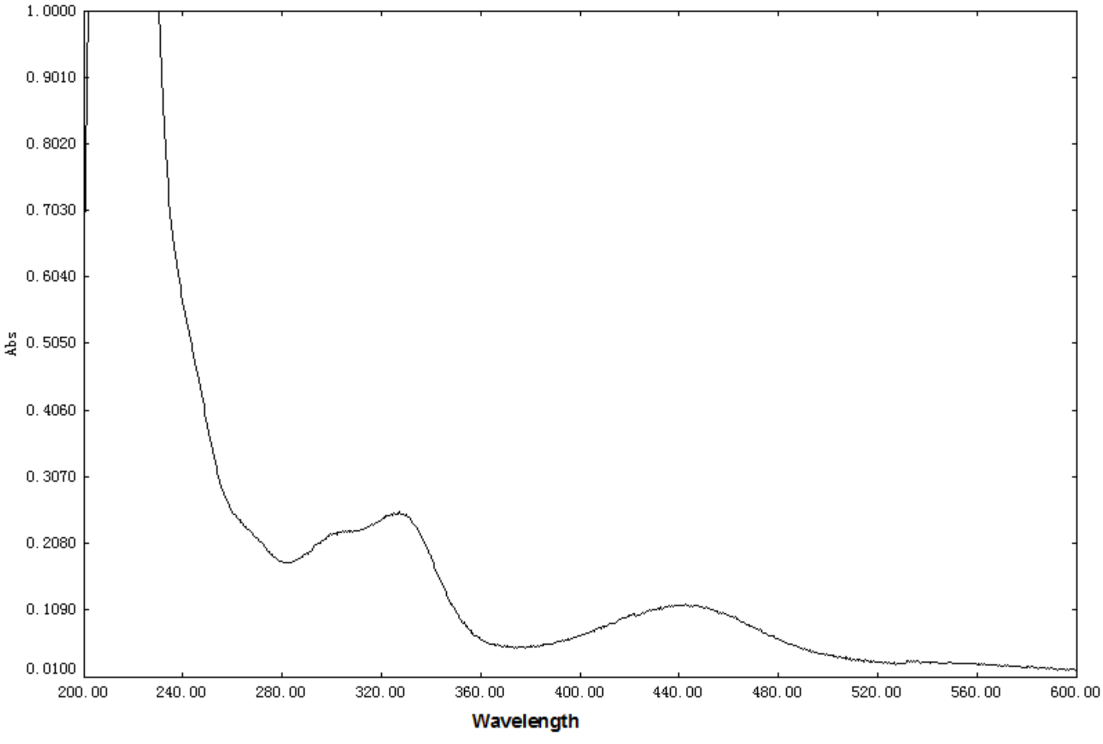


**Figure S22.** UV spectrum of Streptoanthraquinone A (**2**).

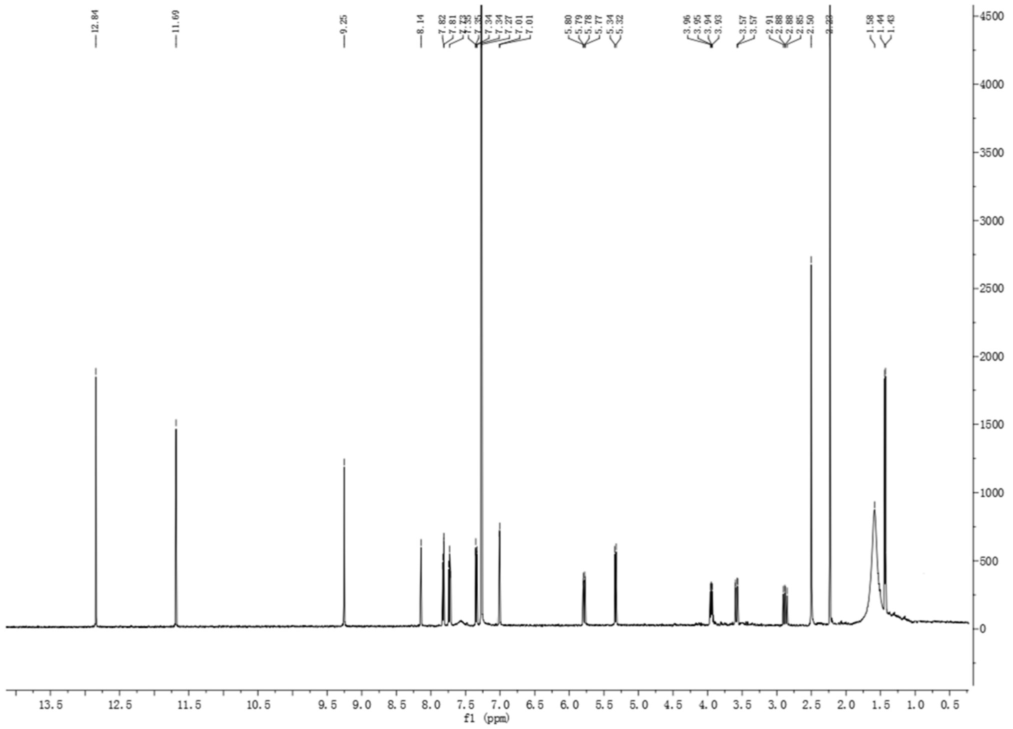

**Figure S23.** 1H-NMR of Streptoanthraquinone A (**2**).

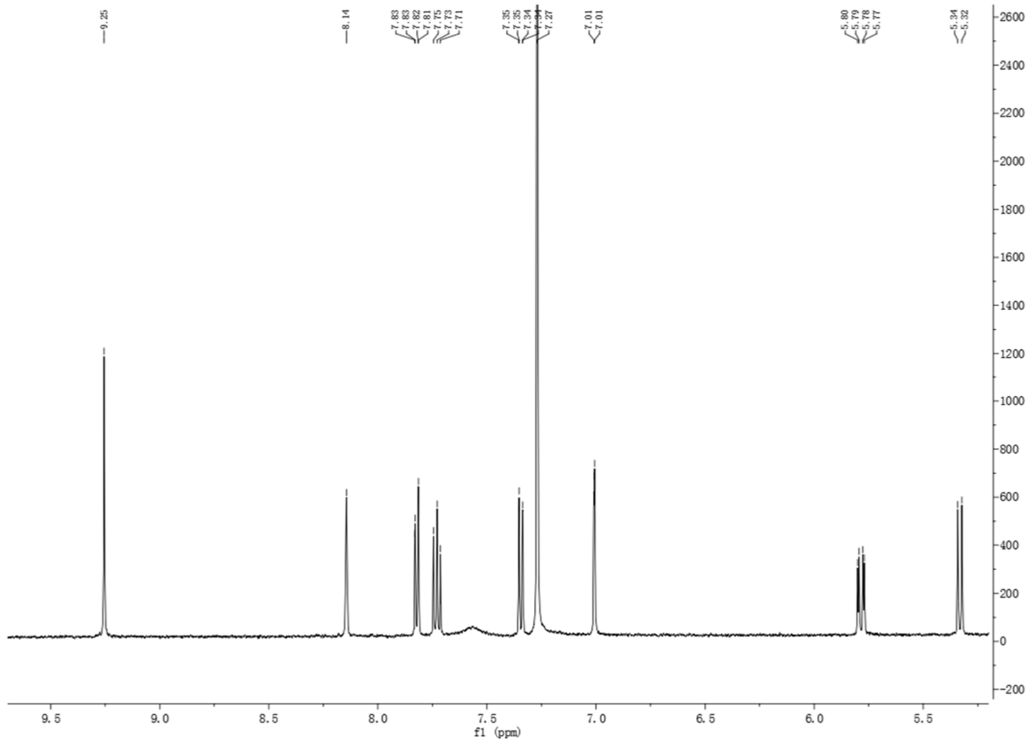

**Figure S24.** 1H-NMR of Streptoanthraquinone A (**2**).

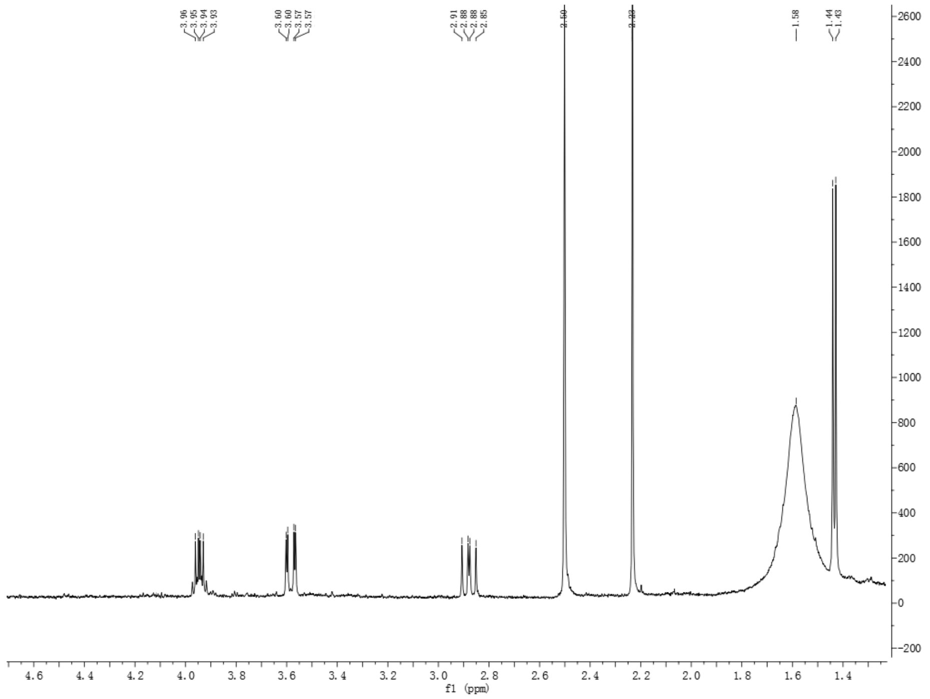


**Figure S25.** 1H-NMR Spectrum of Streptoanthraquinone A (**2**).

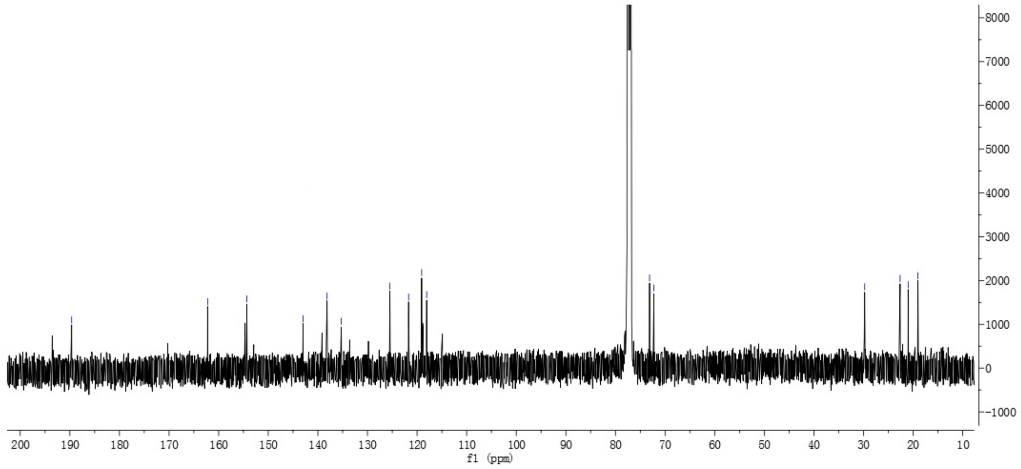


**Figure S26.** 13C-NMR Spectrum of Streptoanthraquinone A (**2**).

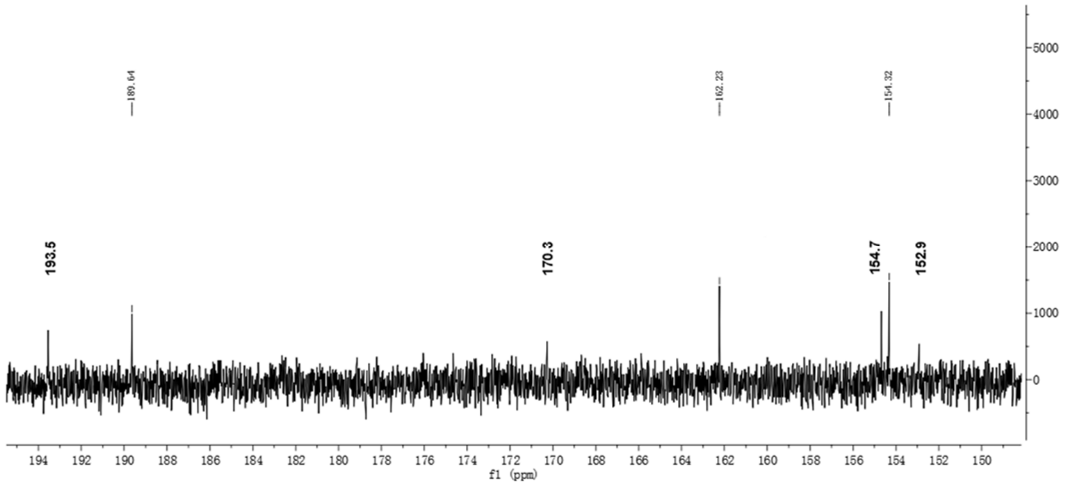


**Figure S27.** 13C-NMR Spectrum of Streptoanthraquinone A (**2**).

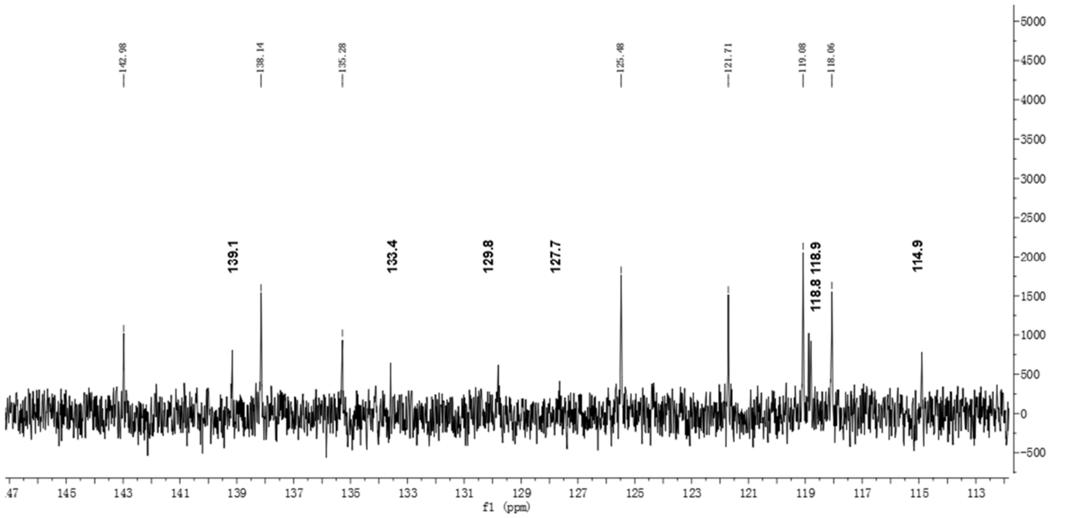


**Figure S28.** 13C-NMR Spectrumof Streptoanthraquinone A (**2**).

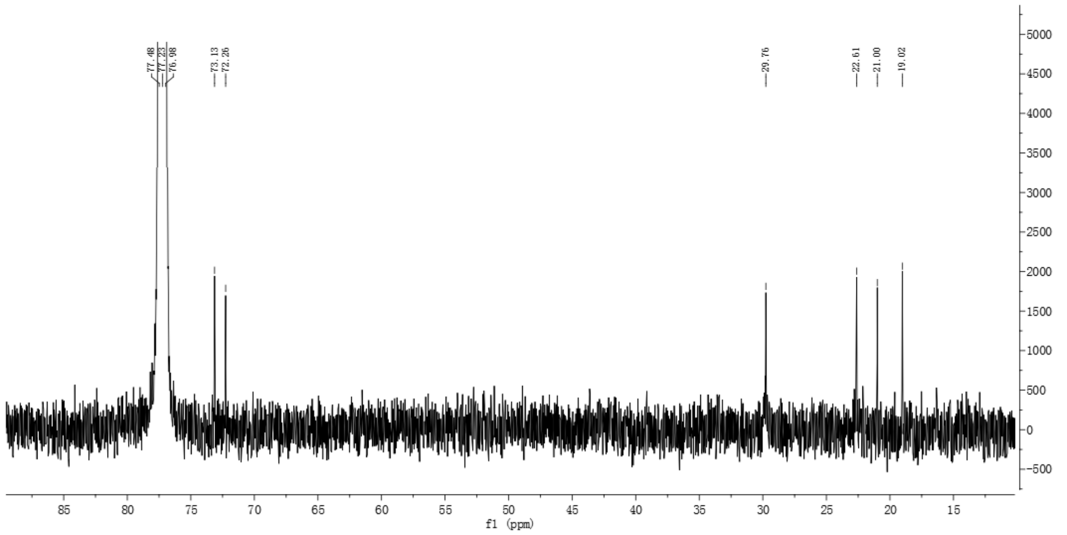


**Figure S29.** 13C-NMR of Streptoanthraquinone A (**2**).

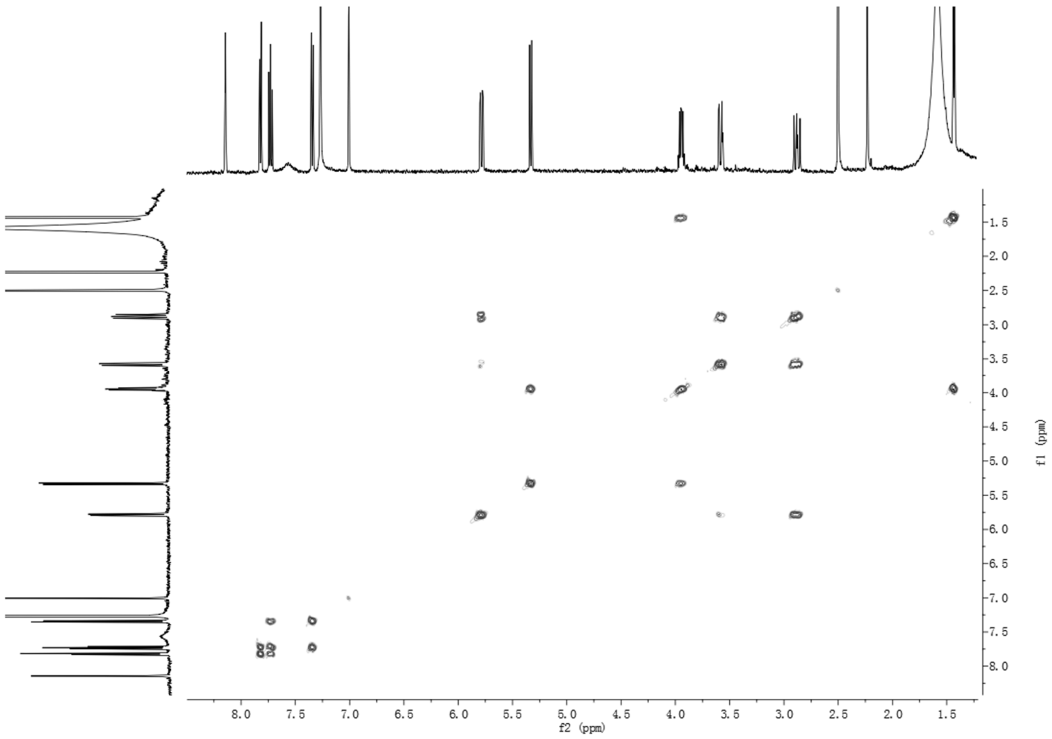


**Figure S30.** 1H-1H COSY of Streptoanthraquinone A (**2**).

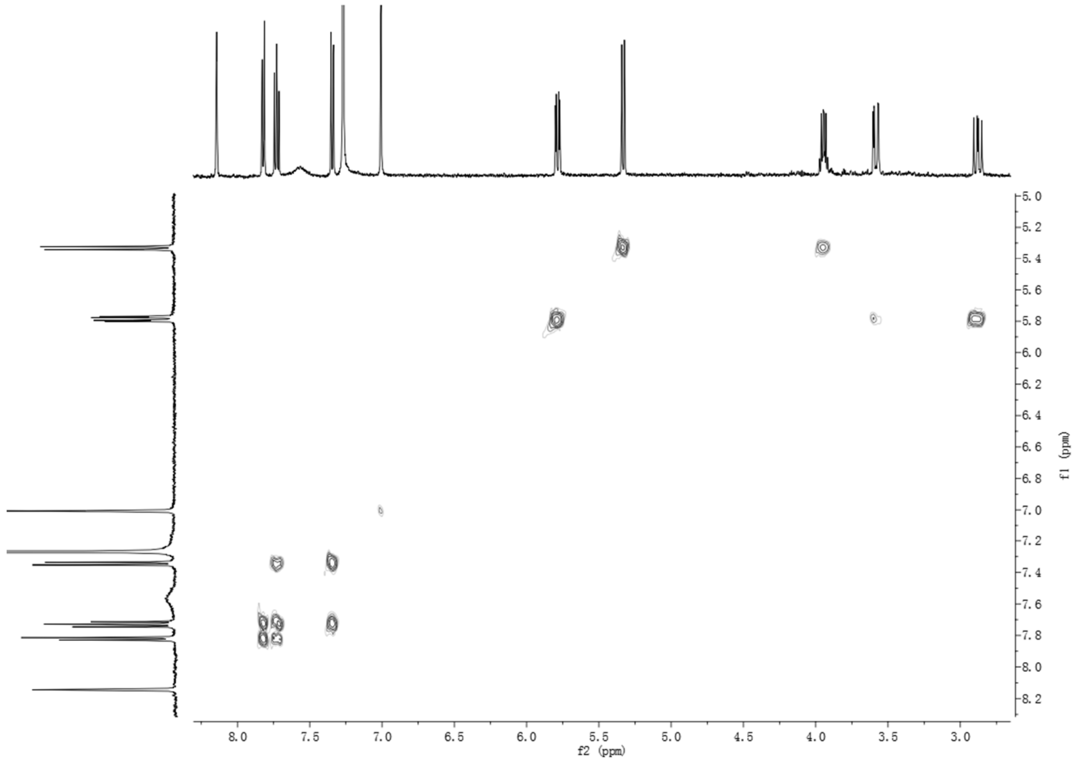


**Figure S31.** 1H-1H COSY of Streptoanthraquinone A (**2**).

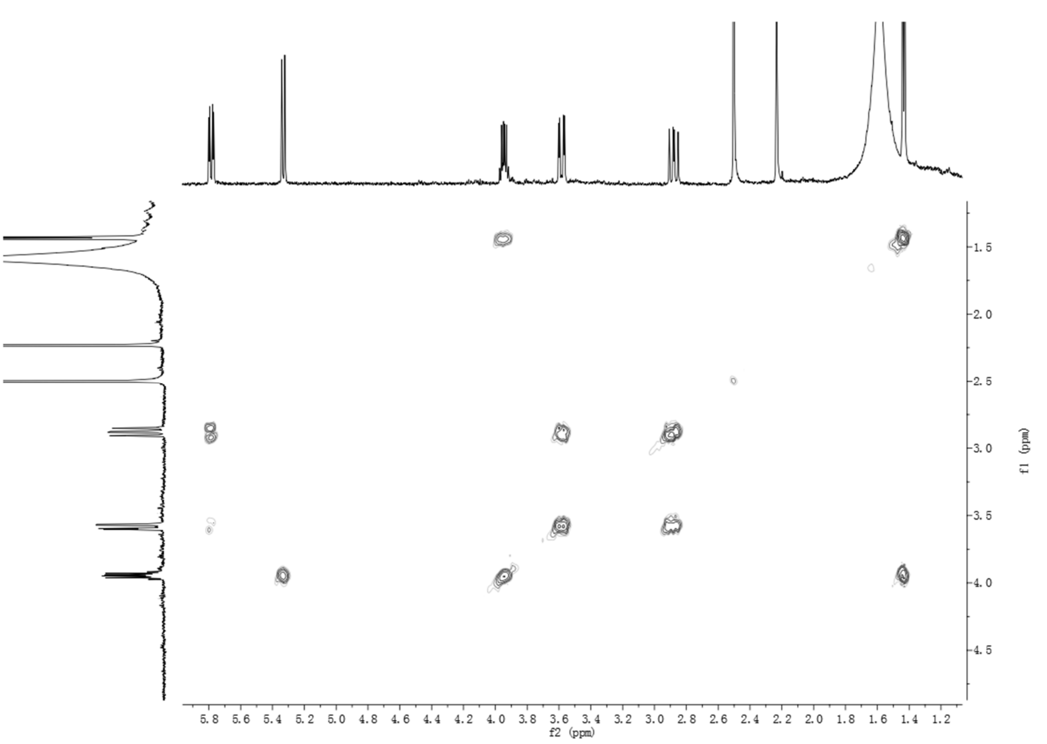


**Figure S32.** 1H-1H COSY of Streptoanthraquinone A (**2**).

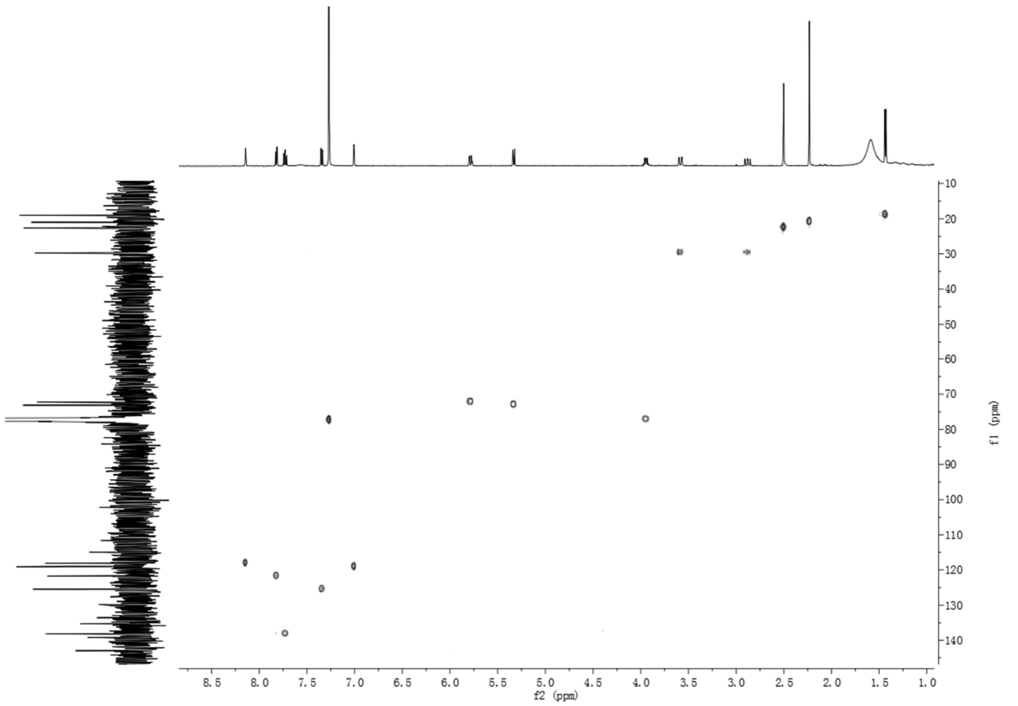


**Figure S33.** HSQC spectrum of Streptoanthraquinone A (**2**).

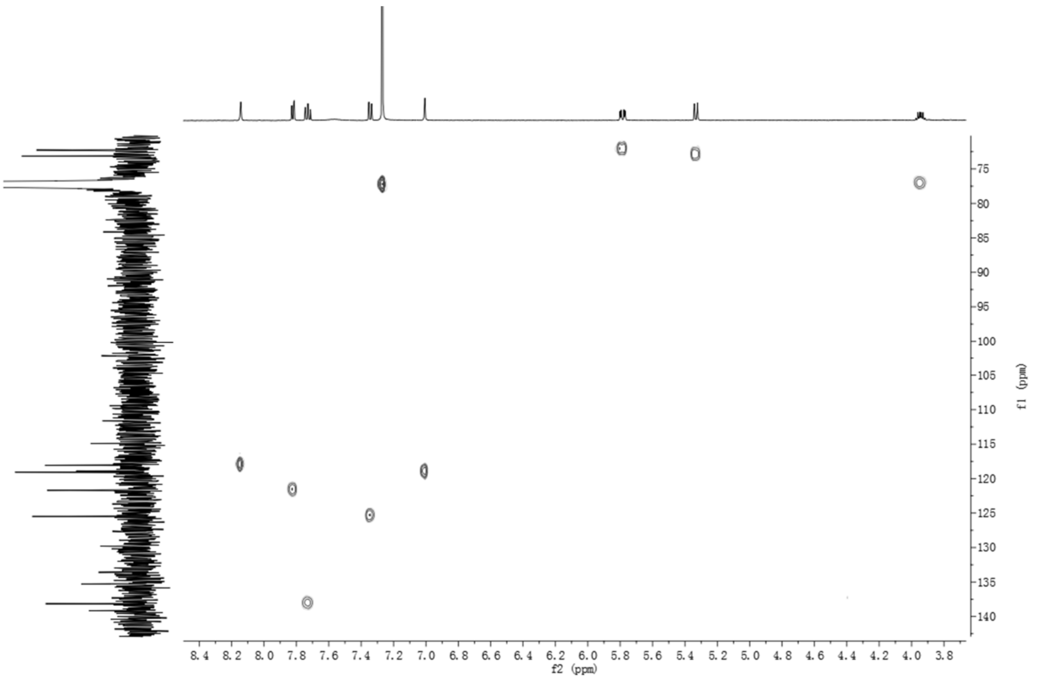


**Figure S34.** HSQC spectrum of Streptoanthraquinone A (**2**).

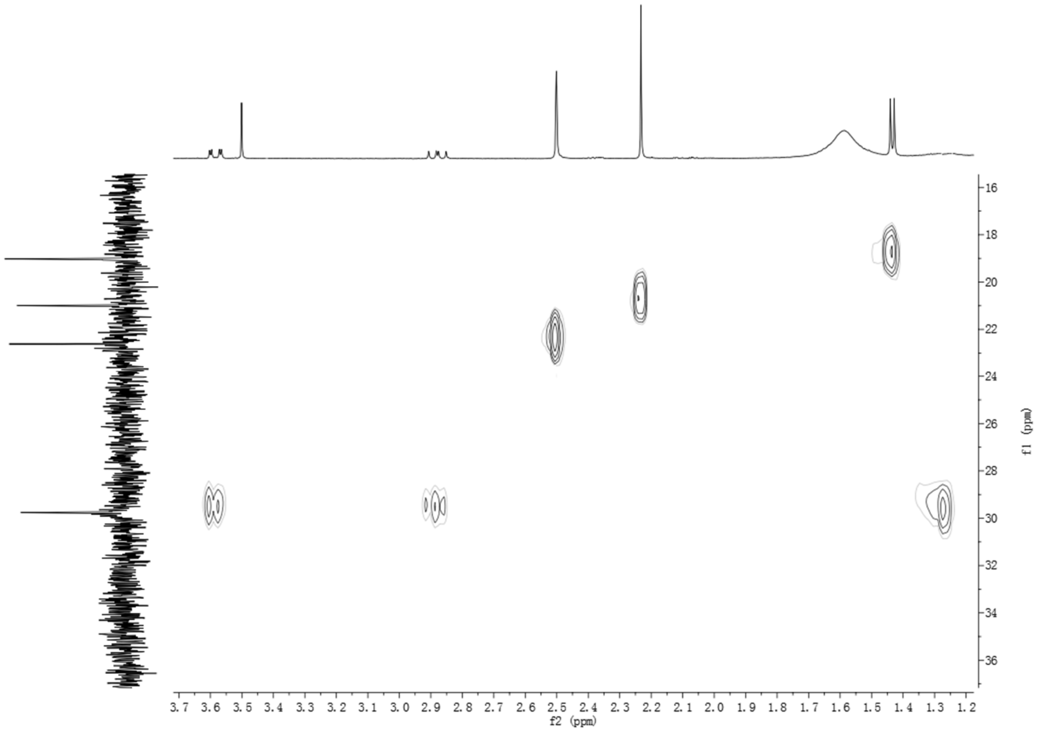


**Figure S35.** HSQC spectrum of Streptoanthraquinone A (**2**).

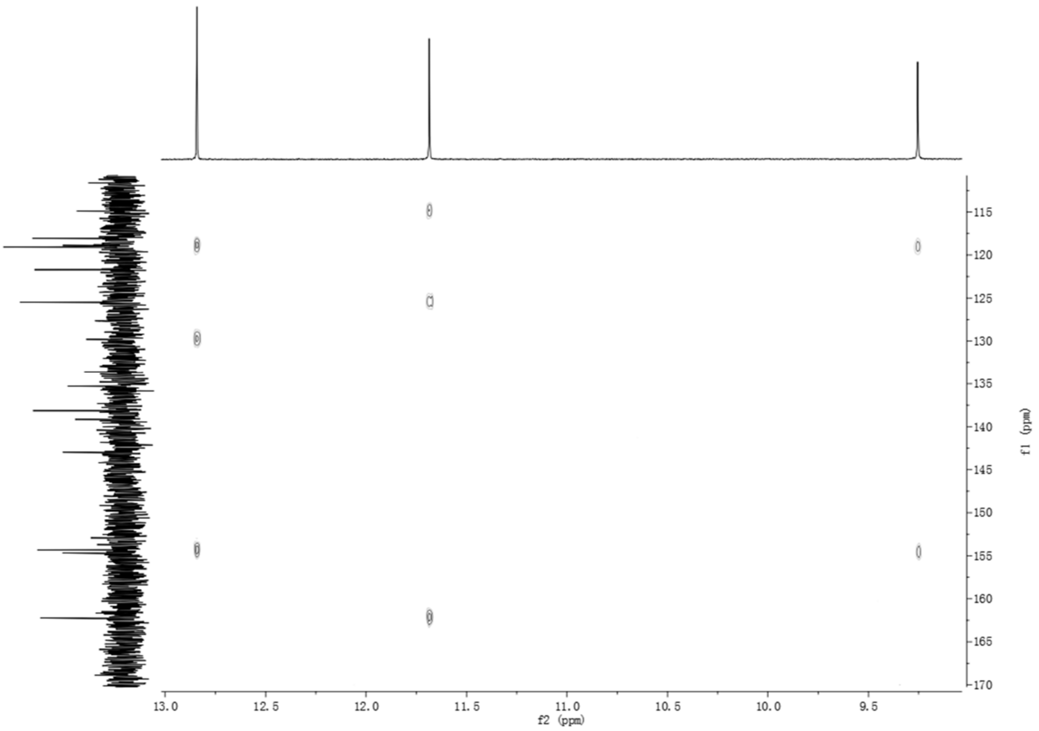


**Figure S36.** HMBC spectrum of Streptoanthraquinone A (**2**).

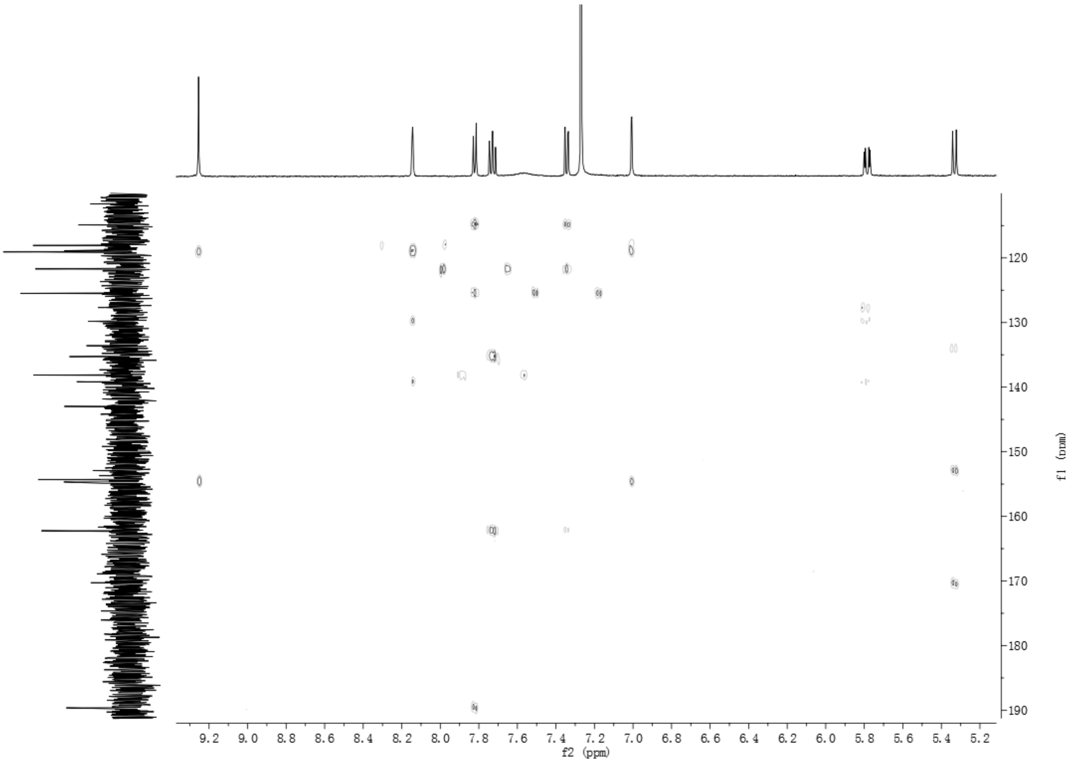


**Figure S37.** HMBC spectrum of Streptoanthraquinone A (**2**).

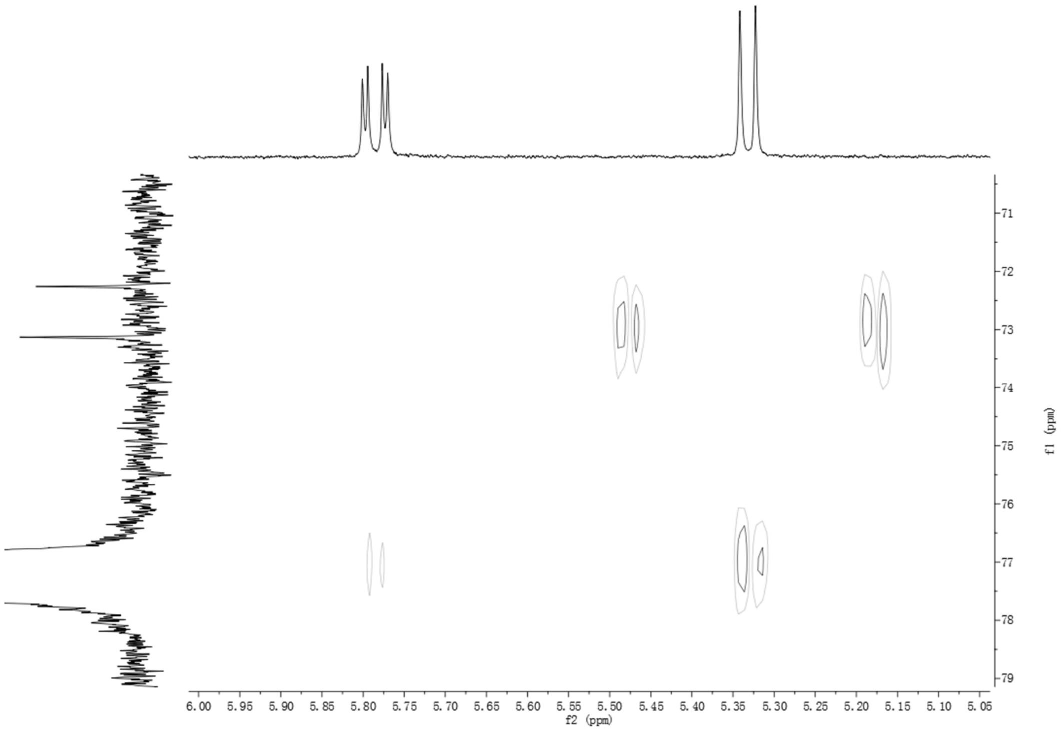


**Figure S38.** HMBC Spectrum of Streptoanthraquinone A (**2**).

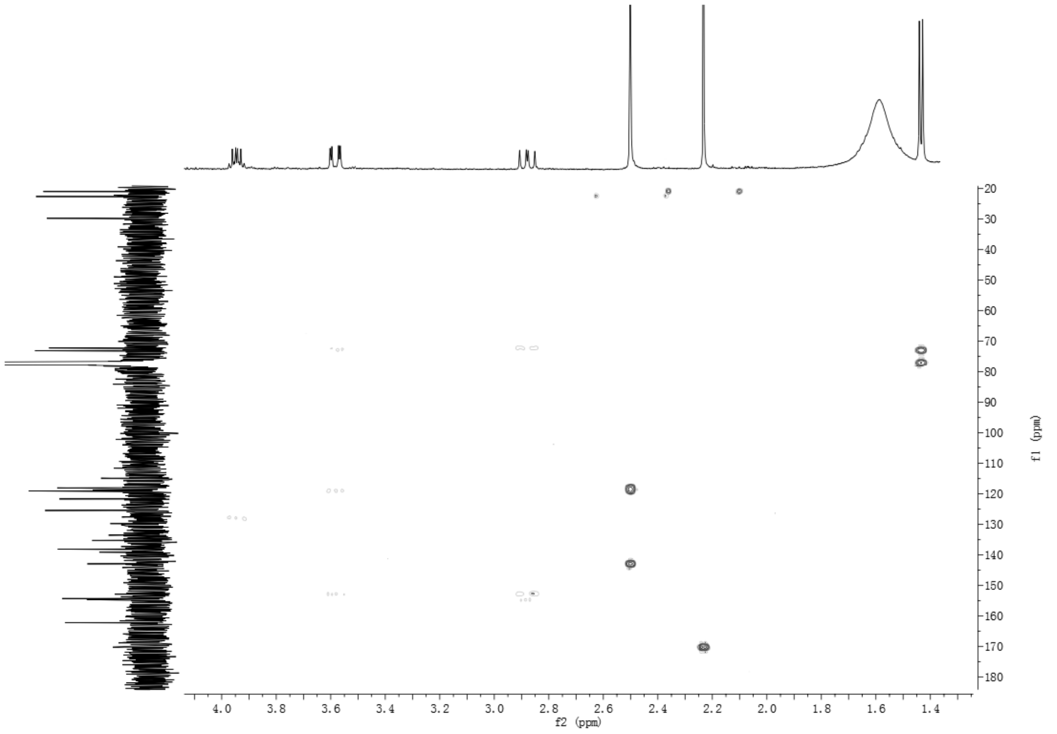


**Figure S39.** HMBC Spectrum of Streptoanthraquinone A (**2**).

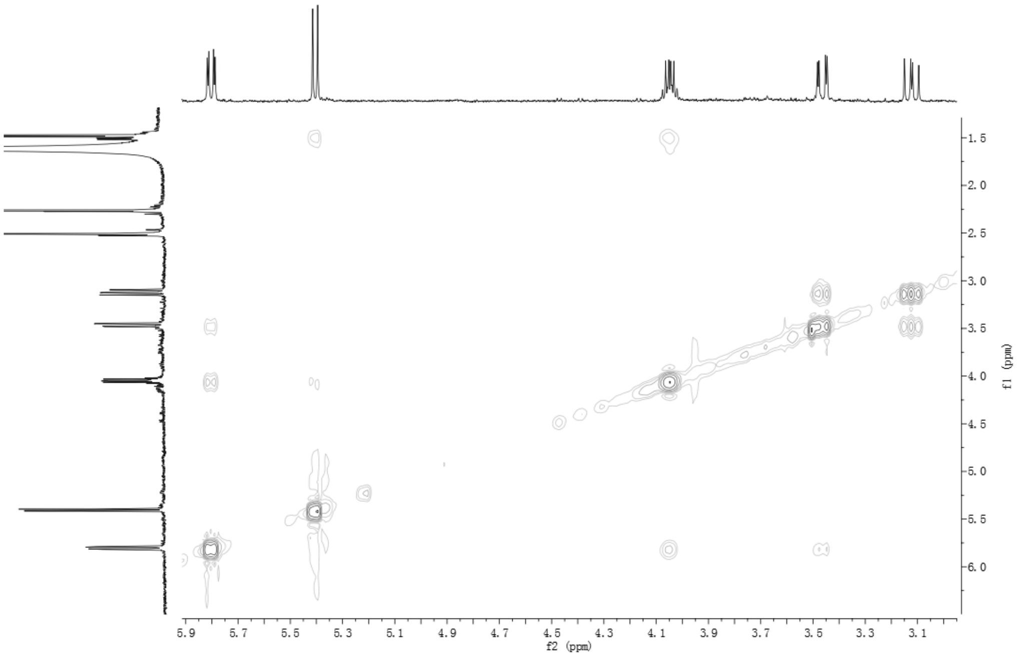

**Figure S40.** NOESY Spectrum of Streptoanthraquinone A (**2**).

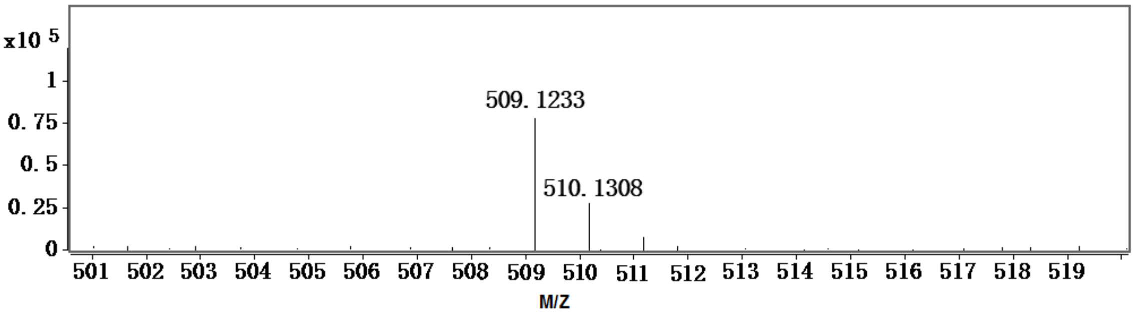


**Figure S41.** HRESIMS Spectrum of Streptoanthraquinone A (**2**).

TGCAGTCGAACGATGAAGCCCTTCGGGGTGGATTAGTGGCGAACGGGTGAGTAACACGT
GGGCAATCTGCCCTTCACTCTGGGACAAGCCCTGGAAACGGGGTCTAATACCGGATAACA
CTCTGTCCCGCATGGGACGGGGTTGAAAGCTCCGGCGGTGAAGGATGAGCCCGCGGCCT
ATCAGCTTGTTGGTGGGGTAATGGCCTACCAAGGCGACGACGGGTAGCCGGCCTGAGAG
GGCGACCGGCCACACTGGGACTGAGACACGGCCCAGACTCCTACGGGAGGCAGCAGTGG
GGAATATTGCACAATGGGCGAAAGCCTGATGCAGCGACGCCGCGTGAGGGATGACGGCC
TTCGGGTTGTAAACCTCTTTCAGCAGGGAAGAAGCGAAAGTGACGGTACCTGCAGAAGA
AGCGCC-GGCTAACTACGTGCCAGCAGCCGCGGTAATACGTAGGGCGCAAGCGTTGTCCG
GAATTATTGGGCGTAAAGAGCTCGTAGGCGGCTTGTCACGTCGGATGTGAAAGCCCGGG
GCTTAACCCCGGGTCTGCATTCGATACGGGCTAGCTAGAGTGTGGTAGGGGAGATCGGAA
TTCCTGGTGTAGCGGTGAAATGCGCAGATATCAGGAGGAACACCGGTGGCGAAGGCGGA
TCTCTGGGCCATTACTGACGCTGAGGAGCGAAAGCGTGGGGAGCGAACAGGATTAGATA
CCCTGGTAGTCCACGCCGTAAACGTTGGGAACTAGGTGTTGGCGACATTCCACGTCGTCG
GTGCCGCAGCTAACGCATTAAGTTCCCCGCCTGGGGAGTACGGCCGCAAGGCTAAAACTC
AAAGGAATTGACGGGGGCCCGCACAAGCAGCGGAGCATGTGGCTTAATTCGACGCAACG
CGAAGAACCTTACCAAGGCTTGACATATACCGGAAAGCATCAGAGATGGTGCCCCCCTTG
TGGTCGGTATACAGGTGGTGCATGG-CTGTCGTCAGCTCGTGTCGTGAGATGTTGGGTTAA
GTCCCGCAACGAGCGCAACCCTTGTTCTGTGTTGCCAGCATGCCCTTCGGGG-TGATGGGG
ACTCACAGGAGACTGCCGGGGTCAACTCGGAGGAAGGTGGGGACGACGTCAAGTCATCA
TGCCCCTTATGTCTTGGGCTGCACACGTGCTACAATGGCCGGTACAATGAGCTGCGATGC
CGCGAGGCGGAGCGAATCTCAAAAAGCCGGTCTCAGTTCGGATTGGGGTCTGCAACTCG
ACCCCATGAAGTCGGAGTTGCTAGTAATCGCAGATCAGCATTGCTGCGGTGAATACGTTC
CCGGGCCTTGTACACACCGCCCGTCACGTCACGAAAGTCGGTAACACCCGAAGCCGGTGG
CCCAACCCC

**Figure S42.** 16S rDNA sequence of *Streptomyces* sp. 182SMLY.

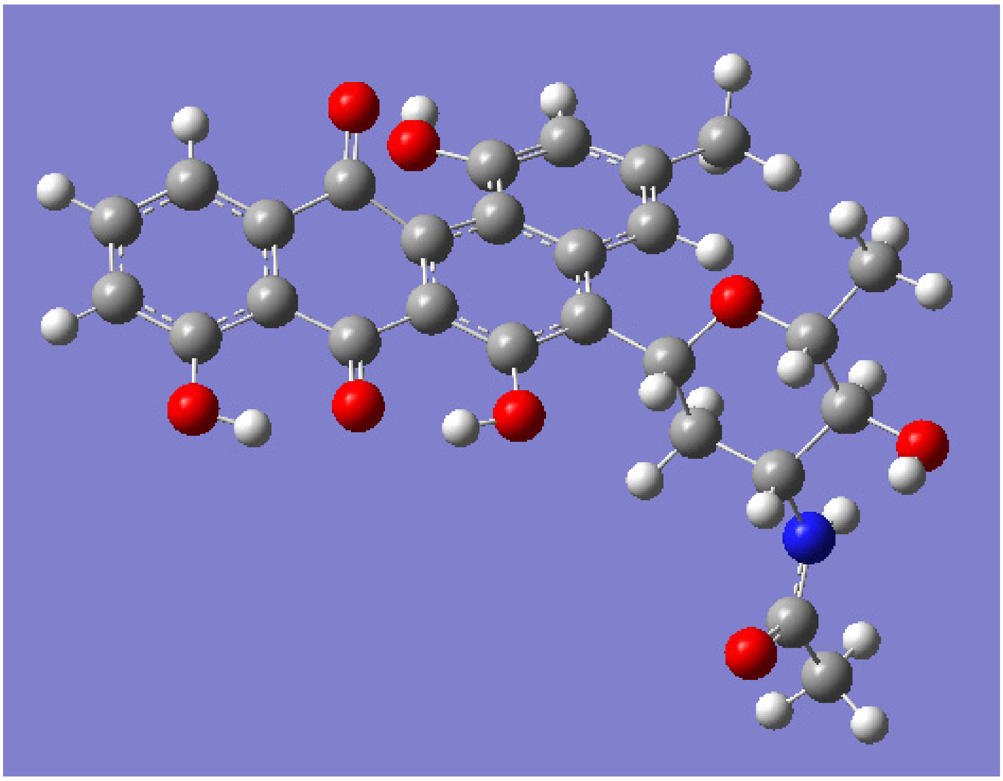

**Figure S43.** The Optimized Geometry of Conformer 1 of (1′*R*, 3′*R*, 4′*S*, 5′*R*)-**1**.

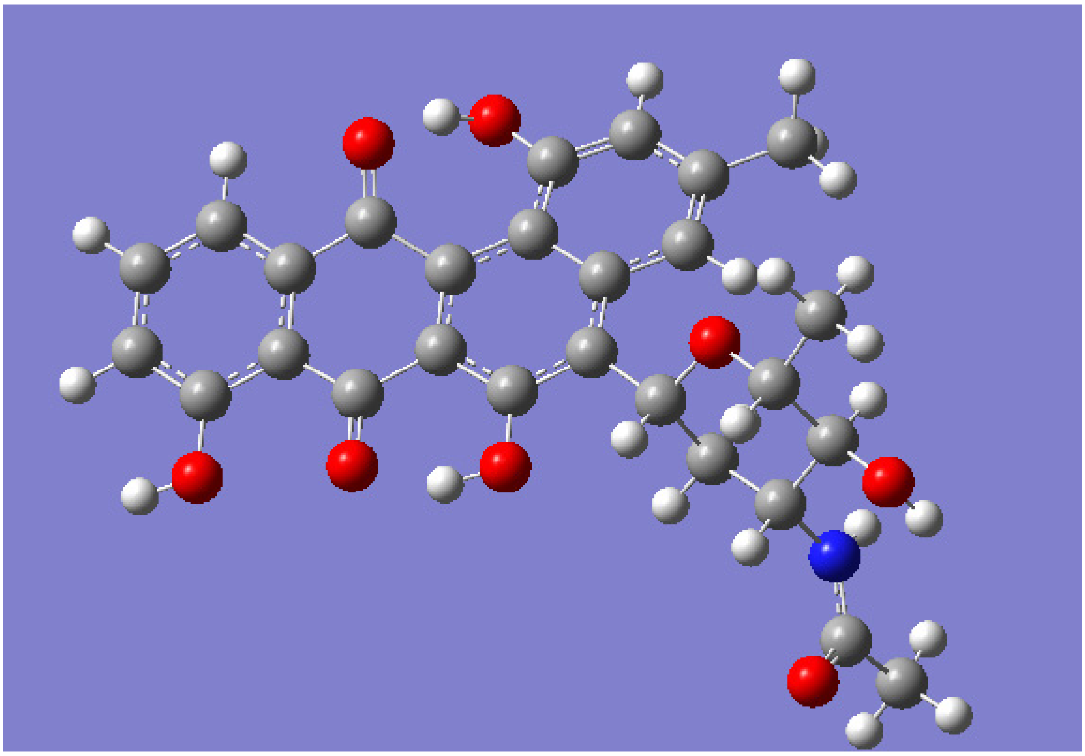

**Figure S44.** The Optimized Geometry of Conformer 2 of (1′*R*, 3′*R*, 4′*S*, 5′*R*)-**1**.

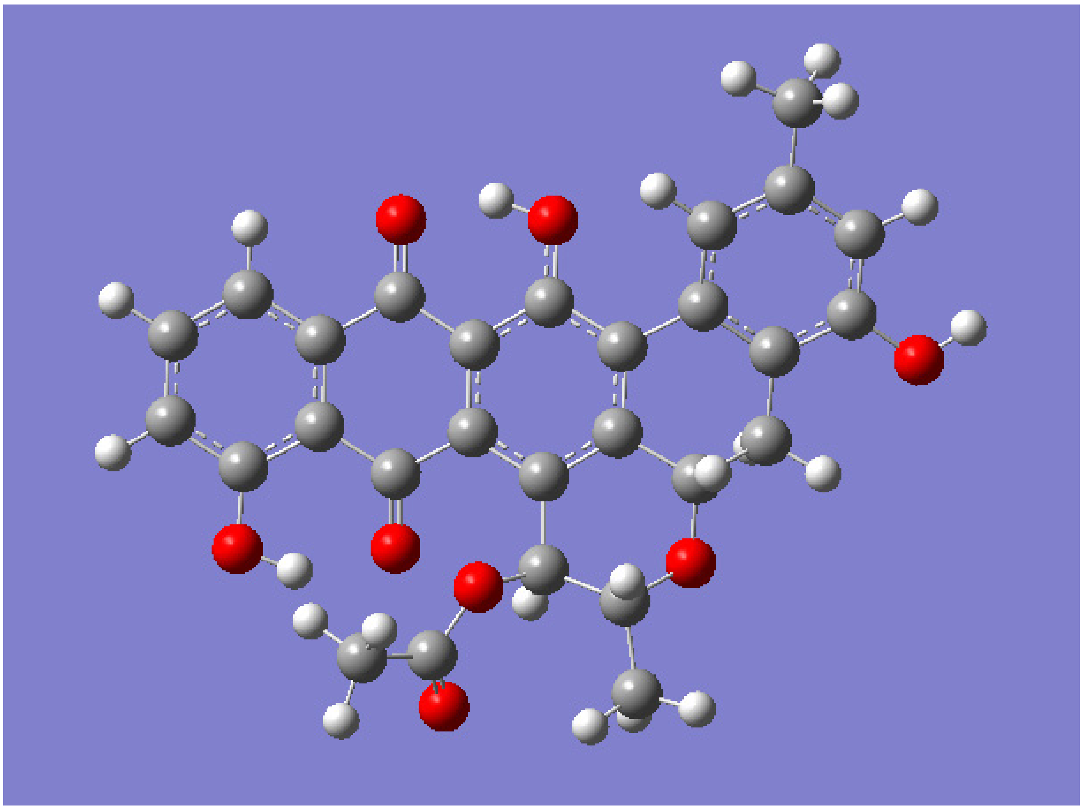

**Figure S45.** The Optimized Geometry of Conformer 1 of (6′*S*, 16′*R*, 17′*R*)-**2**.

**Table S1.** Sequences producing significant alignments.

| Accession | Description | Max Score | Total Score | Query Coverage | Evalue | Ident |
|---|---|---|---|---|---|---|
| KF981731.1 | *Streptomyces griseus* strain CB00830 16S ribosomal RNA gene | 2542 | 2542 | 100% | 0.0 | 100% |
| HQ6074 11.1 | *Streptomyces pluricolorescens* strain 999 16S ribosomal RNA gene | 2542 | 2542 | 100% | 0.0 | 100% |
| FJ792544.1 | *Streptomyces tricolor* strain cfcc3055 16S ribosomal RNA gene | 2542 | 2542 | 100% | 0.0 | 100% |
| EU7814 91.1 | *Streptomyces sporovirgulis* strain L0801 16S ribosomal RNA gene | 2542 | 2542 | 100% | 0.0 | 100% |
| EU257232.1 | *Streptomyces* sp. A15Ydz-AH 16S ribosomal RNA gene | 2542 | 2542 | 100% | 0.0 | 100% |
| EU119184.1 | *Streptomyces* sp. HBUM74775 16S ribosomal RNA gene | 2542 | 2542 | 100% | 0.0 | 100% |
| JQ654447.1 | *Streptomyces sporovirgulis* strain TGNBSA5 16S ribosomal RNA gene | 2540 | 2540 | 99% | 0.0 | 100% |

**Table S2.** Optimized Z-Matrixes of Conformer 1 of (1′*R*, 3′*R*, 4′*S*, 5′*R*)-**1**. Standard orientation.

| Center Number | Atomic Number | Atomic Type | Coordinates (Angstroms) | | |
|---|---|---|---|---|---|
| | | | X | Y | Z |
| 1 | 6 | 0 | −6.57339 | −2.12099 | −0.20611 |
| 2 | 6 | 0 | −5.22477 | −2.43785 | 0.036568 |
| 3 | 6 | 0 | −4.27011 | −1.3966 | 0.13893 |
| 4 | 6 | 0 | −4.70468 | −0.0561 | 0.032749 |
| 5 | 6 | 0 | −6.04365 | 0.247081 | −0.17531 |
| 6 | 6 | 0 | −6.97149 | −0.79675 | −0.31107 |
| 7 | 6 | 0 | −2.86044 | −1.68059 | 0.384568 |
| 8 | 6 | 0 | −3.71602 | 1.038823 | 0.209917 |
| 9 | 6 | 0 | −2.27202 | 0.717085 | 0.000235 |
| 10 | 6 | 0 | −1.87148 | −0.59079 | 0.319624 |
| 11 | 6 | 0 | −0.48253 | −0.86923 | 0.52819 |
| 12 | 6 | 0 | 0.472659 | 0.146009 | 0.478701 |
| 13 | 6 | 0 | 0.090126 | 1.444061 | 0.018538 |
| 14 | 6 | 0 | −1.29802 | 1.711967 | −0.28777 |
| 15 | 1 | 0 | −7.28637 | −2.9349 | −0.29543 |
| 16 | 1 | 0 | −6.35685 | 1.283296 | −0.24217 |
| 17 | 1 | 0 | −8.017 | −0.56637 | −0.49496 |
| 18 | 6 | 0 | −1.59956 | 2.946777 | −0.96337 |
| 19 | 6 | 0 | −0.63525 | 3.91536 | −1.13266 |
| 20 | 1 | 0 | −0.89969 | 4.839408 | −1.64285 |
| 21 | 6 | 0 | 0.699535 | 3.701631 | −0.71189 |
| 22 | 6 | 0 | 1.048479 | 2.479953 | −0.17527 |
| 23 | 1 | 0 | 2.075766 | 2.299137 | 0.105377 |
| 24 | 6 | 0 | 1.717171 | 4.796356 | −0.90129 |
| 25 | 1 | 0 | 1.451636 | 5.68382 | −0.31185 |
| 26 | 1 | 0 | 2.716837 | 4.472867 | −0.59592 |
| 27 | 1 | 0 | 1.764103 | 5.116145 | −1.95009 |
| 28 | 8 | 0 | −4.90258 | −3.73863 | 0.162969 |
| 29 | 1 | 0 | −3.93161 | −3.78506 | 0.353745 |
| 30 | 8 | 0 | −0.06274 | −2.12326 | 0.812715 |
| 31 | 1 | 0 | −0.86411 | −2.71054 | 0.816762 |

**Table S2.** *Cont.*

| Center Number | Atomic Number | Atomic Type | Coordinates (Angstroms) | | |
|---|---|---|---|---|---|
| | | | X | Y | Z |
| 32 | 8 | 0 | −4.07807 | 2.141094 | 0.616494 |
| 33 | 8 | 0 | −2.49044 | −2.86805 | 0.599146 |
| 34 | 6 | 0 | 2.742257 | −0.56084 | −0.44462 |
| 35 | 6 | 0 | 1.90254 | −0.2594 | 0.807303 |
| 36 | 6 | 0 | 4.166068 | −0.98135 | −0.04063 |
| 37 | 1 | 0 | 2.783012 | 0.323388 | −1.09103 |
| 38 | 1 | 0 | 1.853156 | −1.17651 | 1.405948 |
| 39 | 6 | 0 | 4.784373 | 0.042681 | 0.922754 |
| 40 | 1 | 0 | 4.116066 | −1.94762 | 0.473586 |
| 41 | 6 | 0 | 3.826852 | 0.325443 | 2.100374 |
| 42 | 1 | 0 | 4.963172 | 0.984245 | 0.387873 |
| 43 | 1 | 0 | 3.702804 | −0.61233 | 2.669047 |
| 44 | 1 | 0 | 2.273241 | −1.37088 | −1.01425 |
| 45 | 8 | 0 | 2.541593 | 0.742557 | 1.614566 |
| 46 | 6 | 0 | 4.333989 | 1.422545 | 3.022094 |
| 47 | 1 | 0 | 3.63765 | 1.573466 | 3.853869 |
| 48 | 1 | 0 | 5.311092 | 1.151296 | 3.431983 |
| 49 | 1 | 0 | 4.435553 | 2.368802 | 2.477326 |
| 50 | 8 | 0 | 6.065794 | −0.37494 | 1.390407 |
| 51 | 1 | 0 | 5.952001 | −1.22804 | 1.845724 |
| 52 | 7 | 0 | 5.022492 | −1.17524 | −1.20512 |
| 53 | 6 | 0 | 5.237978 | −2.37766 | −1.78539 |
| 54 | 8 | 0 | 4.751606 | −3.43154 | −1.34226 |
| 55 | 6 | 0 | 6.11987 | −2.37871 | −3.01597 |
| 56 | 1 | 0 | 5.562763 | −2.81981 | −3.8497 |
| 57 | 1 | 0 | 6.465406 | −1.38227 | −3.30541 |
| 58 | 1 | 0 | 6.989353 | −3.01848 | −2.82809 |
| 59 | 1 | 0 | 5.419609 | −0.34999 | −1.63877 |
| 60 | 8 | 0 | −2.83922 | 3.09323 | −1.50742 |
| 61 | 1 | 0 | −2.8735 | 3.936539 | −1.99307 |

**Table S3.** Optimized Z-Matrixes of of Conformer 2 of (1′*R*, 3′*R*, 4′*S*, 5′*R*)-**1**. Standard orientation.

| Center Number | Atomic Number | Atomic Type | Coordinates (Angstroms) | | |
|---|---|---|---|---|---|
| | | | X | Y | Z |
| 1 | 6 | 0 | −6.58576 | −2.23913 | −0.21749 |
| 2 | 6 | 0 | −5.22579 | −2.55237 | −0.04242 |
| 3 | 6 | 0 | −4.27982 | −1.50782 | 0.094572 |
| 4 | 6 | 0 | −4.74015 | −0.17343 | 0.055855 |
| 5 | 6 | 0 | −6.09174 | 0.123201 | −0.10331 |
| 6 | 6 | 0 | −7.0112 | −0.91966 | −0.24676 |
| 7 | 6 | 0 | −2.84886 | −1.74223 | 0.311116 |
| 8 | 6 | 0 | −3.78984 | 0.955263 | 0.189873 |
| 9 | 6 | 0 | −2.33905 | 0.722268 | 0.002378 |
| 10 | 6 | 0 | −1.90314 | −0.59576 | 0.252618 |
| 11 | 6 | 0 | −0.51205 | −0.85235 | 0.450178 |
| 12 | 6 | 0 | 0.427728 | 0.17939 | 0.405567 |
| 13 | 6 | 0 | 0.024236 | 1.477156 | −0.02324 |

**Table S3.** *Cont.*

| Center Number | Atomic Number | Atomic Type | Coordinates (Angstroms) | | |
|---|---|---|---|---|---|
| | | | X | Y | Z |
| 14 | 6 | 0 | −1.38345 | 1.768705 | −0.23713 |
| 15 | 6 | 0 | −1.68947 | 3.075246 | −0.77638 |
| 16 | 6 | 0 | −0.69239 | 3.996618 | −1.02366 |
| 17 | 6 | 0 | 0.668964 | 3.718414 | −0.77774 |
| 18 | 6 | 0 | 1.004104 | 2.478981 | −0.28136 |
| 19 | 6 | 0 | 1.71302 | 4.764551 | −1.06972 |
| 20 | 8 | 0 | −4.82076 | −3.84107 | −0.00994 |
| 21 | 8 | 0 | −0.05662 | −2.09646 | 0.709259 |
| 22 | 8 | 0 | −4.24635 | 2.069983 | 0.50507 |
| 23 | 8 | 0 | −2.41961 | −2.89666 | 0.519324 |
| 24 | 6 | 0 | 2.692389 | −0.57965 | −0.50735 |
| 25 | 6 | 0 | 1.860674 | −0.21723 | 0.737271 |
| 26 | 6 | 0 | 4.116116 | −0.96863 | −0.08376 |
| 27 | 6 | 0 | 4.728307 | 0.115567 | 0.820584 |
| 28 | 6 | 0 | 3.790364 | 0.413961 | 2.001742 |
| 29 | 8 | 0 | 2.504198 | 0.811132 | 1.504147 |
| 30 | 6 | 0 | 4.289067 | 1.536586 | 2.897182 |
| 31 | 8 | 0 | 5.977485 | −0.3077 | 1.362452 |
| 32 | 7 | 0 | 4.966122 | −1.22137 | −1.24279 |
| 33 | 6 | 0 | 5.616503 | −2.38936 | −1.4755 |
| 34 | 8 | 0 | 5.586704 | −3.34562 | −0.68646 |
| 35 | 6 | 0 | 6.390619 | −2.46491 | −2.77439 |
| 36 | 8 | 0 | −2.93464 | 3.457681 | −1.17896 |
| 37 | 1 | 0 | −7.29863 | −3.05292 | −0.3242 |
| 38 | 1 | 0 | −6.41542 | 1.15708 | −0.12585 |
| 39 | 1 | 0 | −8.06583 | −0.69909 | −0.38281 |
| 40 | 1 | 0 | −0.99096 | 4.956632 | −1.43617 |
| 41 | 1 | 0 | 2.04132 | 2.251979 | −0.08619 |
| 42 | 1 | 0 | 1.520871 | 5.682519 | −0.49946 |
| 43 | 1 | 0 | 2.717322 | 4.411127 | −0.81706 |
| 44 | 1 | 0 | 1.705114 | 5.043858 | −2.13126 |
| 45 | 1 | 0 | −5.59062 | −4.42412 | −0.14314 |
| 46 | 1 | 0 | −0.85518 | −2.69767 | 0.712807 |
| 47 | 1 | 0 | 2.722391 | 0.270943 | −1.1987 |
| 48 | 1 | 0 | 1.808763 | −1.11037 | 1.370678 |
| 49 | 1 | 0 | 4.090491 | −1.90175 | 0.486687 |
| 50 | 1 | 0 | 4.858397 | 1.041254 | 0.237173 |
| 51 | 1 | 0 | 3.677078 | −0.51135 | 2.589953 |
| 52 | 1 | 0 | 2.219512 | −1.41776 | −1.03058 |
| 53 | 1 | 0 | 3.577903 | 1.714537 | 3.711067 |
| 54 | 1 | 0 | 5.256375 | 1.273315 | 3.334766 |
| 55 | 1 | 0 | 4.404831 | 2.465867 | 2.326833 |
| 56 | 1 | 0 | 6.56145 | −0.51664 | 0.613089 |
| 57 | 1 | 0 | 6.353254 | −1.53951 | −3.35525 |
| 58 | 1 | 0 | 7.435235 | −2.70826 | −2.55295 |
| 59 | 1 | 0 | 5.984073 | −3.28311 | −3.37934 |
| 60 | 1 | 0 | 5.015243 | −0.49768 | −1.95105 |
| 61 | 1 | 0 | −3.59531 | 3.090039 | −0.54117 |

**Table S4.** Optimized Z-Matrixes of Conformer 1 of (6′*S*, 16′*R*, 17′*R*)-**2**. Standard orientation.

| Center Number | Atomic Number | Atomic Type | Coordinates (Angstroms) | | |
|---|---|---|---|---|---|
| | | | X | Y | Z |
| 1 | 6 | 0 | 5.715434 | −2.26822 | −0.43924 |
| 2 | 6 | 0 | 4.907718 | −1.12172 | −0.32309 |
| 3 | 6 | 0 | 3.495798 | −1.25714 | −0.24673 |
| 4 | 6 | 0 | 2.941269 | −2.55743 | −0.27372 |
| 5 | 6 | 0 | 3.752335 | −3.68344 | −0.38148 |
| 6 | 6 | 0 | 5.141711 | −3.52954 | −0.4691 |
| 7 | 6 | 0 | 2.640604 | −0.07112 | −0.13037 |
| 8 | 6 | 0 | 1.479828 | −2.72632 | −0.17092 |
| 9 | 6 | 0 | 0.615796 | −1.54209 | −0.11636 |
| 10 | 6 | 0 | 1.158195 | −0.22385 | −0.14482 |
| 11 | 6 | 0 | 0.281076 | 0.882229 | −0.19481 |
| 12 | 6 | 0 | −1.11021 | 0.646101 | −0.21102 |
| 13 | 6 | 0 | −1.67063 | −0.62528 | −0.03838 |
| 14 | 6 | 0 | −0.78923 | −1.7372 | −0.05084 |
| 15 | 6 | 0 | −3.14315 | −0.76589 | 0.092731 |
| 16 | 6 | 0 | −2.04477 | 1.817436 | −0.458 |
| 17 | 6 | 0 | −3.29621 | 1.381253 | −1.21226 |
| 18 | 6 | 0 | −3.94768 | 0.224891 | −0.50216 |
| 19 | 6 | 0 | −3.74617 | −1.80542 | 0.826174 |
| 20 | 6 | 0 | −5.34207 | 0.114805 | −0.40165 |
| 21 | 6 | 0 | −5.13518 | −1.89043 | 0.94864 |
| 22 | 6 | 0 | −5.93157 | −0.92655 | 0.317593 |
| 23 | 8 | 0 | −6.09359 | 1.075216 | −1.03093 |
| 24 | 8 | 0 | −1.3229 | −2.96388 | −0.04223 |
| 25 | 8 | 0 | 5.528483 | 0.070101 | −0.30513 |
| 26 | 8 | 0 | 3.175277 | 1.052955 | −0.02305 |
| 27 | 6 | 0 | 0.765313 | 2.32821 | −0.16955 |
| 28 | 6 | 0 | −0.30355 | 3.387558 | −0.5613 |
| 29 | 8 | 0 | 1.167581 | 2.579442 | 1.217536 |
| 30 | 6 | 0 | 2.253035 | 3.341509 | 1.453634 |
| 31 | 6 | 0 | 2.611676 | 3.334517 | 2.915187 |
| 32 | 8 | 0 | 2.859282 | 3.9532 | 0.591719 |
| 33 | 8 | 0 | −1.38358 | 2.78215 | −1.26389 |
| 34 | 6 | 0 | 0.24281 | 4.482099 | −1.46527 |
| 35 | 8 | 0 | 1.000038 | −3.88376 | −0.15981 |
| 36 | 6 | 0 | −5.77549 | −3.0129 | 1.730495 |
| 37 | 1 | 0 | 6.790955 | −2.13312 | −0.50554 |
| 38 | 1 | 0 | 3.302318 | −4.66933 | −0.39649 |
| 39 | 1 | 0 | 5.776606 | −4.40639 | −0.55875 |
| 40 | 1 | 0 | −3.00473 | 1.103875 | −2.23643 |
| 41 | 1 | 0 | −3.13125 | −2.54733 | 1.319522 |
| 42 | 1 | 0 | −7.01691 | −0.97613 | 0.390084 |
| 43 | 1 | 0 | −7.0365 | 0.910262 | −0.85759 |
| 44 | 1 | 0 | −0.56319 | −3.61983 | −0.09372 |
| 45 | 1 | 0 | 4.815587 | 0.760657 | −0.21666 |
| 46 | 1 | 0 | 1.748047 | 3.624039 | 3.523736 |
| 47 | 1 | 0 | 2.90227 | 2.320316 | 3.212403 |
| 48 | 1 | 0 | 3.44249 | 4.019933 | 3.093852 |

**Table S4.** *Cont.*

| Center Number | Atomic Number | Atomic Type | Coordinates (Angstroms) | | |
|---|---|---|---|---|---|
| | | | X | Y | Z |
| 49 | 1 | 0 | 1.647865 | 2.445419 | −0.79431 |
| 50 | 1 | 0 | −0.69079 | 3.824665 | 0.371479 |
| 51 | 1 | 0 | 0.641248 | 4.053682 | −2.39233 |
| 52 | 1 | 0 | −0.55699 | 5.184946 | −1.72352 |
| 53 | 1 | 0 | 1.044201 | 5.027378 | −0.96094 |
| 54 | 1 | 0 | −3.97812 | 2.231517 | −1.28916 |
| 55 | 1 | 0 | −2.33394 | 2.275675 | 0.503258 |
| 56 | 1 | 0 | −6.53505 | −2.6356 | 2.425838 |
| 57 | 1 | 0 | −6.2785 | −3.72513 | 1.062556 |
| 58 | 1 | 0 | −5.03038 | −3.56936 | 2.308534 |

© 2016 by the authors; licensee MDPI, Basel, Switzerland. This article is an open access article distributed under the terms and conditions of the Creative Commons by Attribution (CC-BY) license (http://creativecommons.org/licenses/by/4.0/).
